# Modulation of Polyunsaturated Fatty Acid Production and Nutritional Lipid Quality Indices in *Rhodotorula mucilaginosa* 6S Using Molasses as a Sustainable Carbon Source

**DOI:** 10.3390/biom16091294

**Published:** 2026-09-08

**Authors:** Ivis Páez, Edgar Uquiche, Adalberto Pessoa, Robinson Soto-Ramirez, Paola Díaz-Navarrete

**Affiliations:** 1 Doctorado en Ciencias de la Ingeniería mención Bioprocesos, Universidad de La Frontera, Av. Francisco Salazar 01145, Temuco 4811230, Chile; i.paez02@ufromail.cl; 2 Departamento de Ingeniería Química, Center of Food Biotechnology and Bioseparation, Universidad de La Frontera, BIOREN, Av. Francisco Salazar 01145, Temuco 4811230, Chile; edgar.uquiche@ufrontera.cl; 3 Department of Pharmaceutical-Biochemical Technology, School of Pharmaceutical Sciences, University of Saõ Paulo, Saõ Paulo 4030000, Brazil; pessoajr@usp.br; 4 Departamento de Ingeniería de Procesosy Bioproductos, Facultad de Ingeniería, Universidad del Bío-Bío, Avda Collao. 1202, Concepción 4051381, Chile; rdsoto@ubiobio.cl; 5 Departamento de Ciencias Veterinarias y Salud Pública, Facultad de Recursos Naturales, Universidad Católica de Temuco, Temuco 4780000, Chile

**Keywords:** oleaginous yeasts, *Rhodotorula mucilaginosa*, polyunsaturated lipids, molasses, lipid nutritional indices

## Abstract

Microbial lipids produced by oleaginous yeasts are a sustainable alternative to conventional lipid sources because of their ability to generate nutritionally valuable fatty acids. This study evaluated the effects of carbon-to-nitrogen (C/N) ratio, modified through molasses concentration, and cultivation temperature (15 and 25 °C) on biomass production, lipid accumulation, fatty acid composition, and nutritional quality of lipids produced by the native yeast *Rhodotorula mucilaginosa* 6S. Biomass, lipids, sugar consumption, and growth kinetics were determined, while fatty acid composition was analyzed by gas chromatography and nutritional quality was assessed through lipid nutritional indices. Biomass production was mainly influenced by the C/N ratio, whereas temperature had the greatest effect on lipid accumulation and fatty acid composition. Cultivation at 25 °C enhanced lipid yield and productivity, while 15 °C promoted faster growth and higher accumulation of polyunsaturated fatty acids (PUFAs), resulting in lipids with superior nutritional quality. The combination of 15 °C and a C/N ratio of 12 produced the most favorable lipid profile, with higher unsaturation, improved PUFA/SFA and MUFA/SFA ratios, balanced ω-6/ω-3 and LA/ALA ratios, low atherogenicity and thrombogenicity indices, and a high hypocholesterolemic/hypercholesterolemic ratio. These findings demonstrate the potential of molasses as a sustainable substrate for producing high-quality microbial lipids.

## 1. Introduction

Polyunsaturated fatty acids (PUFAs) play a fundamental role in human and animal nutrition, participating in essential physiological processes such as the regulation of cellular metabolism, the structure of biological membranes, and the modulation of the inflammatory response [1,2]. In particular, omega-3 and omega-6 fatty acids are modulators of gene expression and are directly related to the prevention and progression of chronic diseases such as Parkinson’s and Alzheimer’s [3,4]. Humans cannot synthesize omega-3 fatty acids, making their intake through diet indispensable [5]. Traditionally, these compounds are obtained from fish oils and vegetable oils, whose production presents increasing environmental, economic and sustainability questions [6].

In this context, oleaginous microorganisms have emerged as an alternative for the sustainable production of lipids and PUFAs [7]. Among them, oleaginous yeasts stand out for their rapid growth, their ability to adapt to adverse environmental conditions, and their potential to utilize low-cost substrates, including agro-industrial waste [8]. Several species can accumulate more than 20% of their dry weight as lipids, with fatty acid profiles that can be tailored by modifying cultivation conditions [9,10]. Therefore, the potential of oleaginous yeasts is not restricted to maximizing total lipid accumulation, but also includes the possibility of modulating lipid composition according to the cultivation environment.

Within this group, the genus *Rhodotorula* has received special attention due to its high lipid accumulation capacity, metabolic versatility, and ability to produce high-value compounds such as triacylglycerols, phospholipids, sterols, and carotenoids. Previous research has reported lipid contents ranging from 24 to 70% of cell dry weight, depending on the species and culture conditions, as well as lipid profiles rich in saturated, monounsaturated, and polyunsaturated fatty acids [9]. For example, *Rhodotorula pacifica* INDKK and *Rhodotorula graminis* have been reported to accumulate 57.84% and 29.69% total lipids, respectively [11,12]. These results demonstrate the metabolic potential of the genus, while also highlighting that lipid accumulation and fatty acid composition are strongly dependent on the cultivation conditions rather than being fixed characteristics of the microorganism.

*Rhodotorula mucilaginosa* 6S is particularly relevant in this context [13]. Previous study with this strain reported a lipid profile composed mainly of saturated fatty acids (SAFA, 47.6%), followed by polyunsaturated fatty acids (PUFA, 29.6%) and monounsaturated fatty acids (MUFA, 24%). However, when the yeast was cultured with 30 mg/L of Na_2_SeO_3_ for 48 h, the profile changed significantly to: SAFA, 22.0%; MUFA, 69.5%; and PUFA, 8.2% [14]. The fatty acid composition of this strain can be substantially modified by environmental conditions, indicating that cultivation parameters may influence not only the amount of lipid produced but also its potential nutritional and functional value. Temperature is especially relevant because changes in membrane fluidity associated with environmental temperature can alter the degree of fatty acid unsaturation, with lower temperatures generally favoring a greater proportion of unsaturated fatty acids [5]. Similarly, carbon availability relative to nitrogen availability is a key determinant of the balance between cellular growth and lipid metabolism in oleaginous yeasts. Nitrogen limitation in the presence of excess carbon can promote lipid accumulation, reaching up to 40–70% of dry cell weight, whereas less restrictive nitrogen conditions tend to favor biomass formation [15]. Previous studies on *Rhodosporidium kratochvilovae* suggest that a large amount of neutral lipids accumulate when grown under nitrogen-limited conditions [16]. Thus, the carbon-to-nitrogen (C/N) ratio provides a direct means of examining the metabolic balance between biomass production and lipid synthesis.

The type of carbon source is another key factor of microbial lipid production, using both pure substrates (glucose, xylose, glycerol) and renewable raw materials and agro-industrial byproducts [9]. In *Rhodotorula glutinis* cultivated in industrial vinasse (30 °C, pH 7.0, and C/N ratio = 80), 48.75% SAFAs have been reported, consisting mainly of palmitic acid C16:0 (34.83%), stearic acid C18:0 (5.83%), and myristic acid C14:0 (4.66%). The MUFAs, comprising 38.3%, are mainly composed of oleic acid C18:1 (30.83%) and heptadecenoic acid C17:1 (3.4%). In PUFAs, 6.37% of C18:2 linoleic acid accumulated [6]. Additionally, aeration, pH, medium composition, oxidative stress, and incubation time influence both lipid content and fatty acid profile. Furthermore, culture strategies such as feed-batch cultivation allow for the control of nutrient availability and improved lipid production, which has been previously reported in yeasts such as *Rhodosporidium toruloides* [17].

A key aspect for improving the viability of these processes is the valorization of agro-industrial byproducts, which contributes to the circular economy approach and the reduction in environmental impact [17,18]. In this regard, molasses is presented as an attractive substrate due to its high sugar content, wide availability, and low cost [19]. Its use therefore provides an opportunity to investigate whether a complex and low-cost carbon source can simultaneously support microbial biomass formation and lipid production. Previous studies have demonstrated the capacity of oleaginous yeasts, including *Rhodosporidium toruloides* and *Rhodotorula glutinis*, to grow efficiently on molasses and reach lipid contents of 53% and 45%, respectively [20]. However, beyond maximizing lipid production, it is also essential to evaluate the nutritional quality of the lipids obtained, as their fatty acid composition ultimately determines their potential value for food and feed applications.

Nutritional fatty acid indices are relevant tools for evaluating the functional quality and potential health impact of a lipid fraction. This is because dietary fats are composed of mixtures of saturated, monounsaturated, and polyunsaturated fatty acids, which can exert beneficial or detrimental effects depending on their proportion and type. Beyond classic indicators such as total SFA, MUFA, and PUFA content, or the ω-6/ω-3 ratio, indices such as PUFA/SFA, atherogenic index (AI), thrombogenic index (TI), hypocholesterolemic/hypercholesterolemic ratio (HH), degree of unsaturation (DU), LA/ALA, and EPA + DHA allow for a more integrated assessment of lipid quality. In particular, AI and TI are widely used because they provide direct information on the potential effect of fatty acids on processes associated with cardiovascular risk. Likewise, the HH ratio allows estimation of the balance between fatty acids with hypocholesterolemic and hypercholesterolemic effects, while the LA/ALA ratio and the EPA + DHA content are especially relevant to assess the balance between essential fatty acids and the nutritional quality of lipids [21,22,23]. Considering these parameters together with biomass and total lipid production allows the metabolic response of the yeast to be evaluated from both a production and a quality perspective.

Given the growing interest in sustainable alternatives for the production of microbial lipids and polyunsaturated fatty acids, *Rhodotorula mucilaginosa* 6S has emerged as a promising oleaginous yeast due to its ability to accumulate lipids and utilize low-cost agro-industrial substrates, such as molasses. Valorizing these byproducts contributes to improving the economic viability and sustainability of bioprocesses. Furthermore, cultivation strategies and operating conditions significantly influence biomass and lipid production, as well as the fatty acid profile, which determines its potential applications in areas such as biodiesel, nutrition, and aquaculture. This study evaluated the effects of C/N ratio and cultivation temperature on biomass production, lipid accumulation, and lipid composition in *Rhodotorula mucilaginosa* 6S grown on molasses. Fatty acid profiles and nutritional lipid quality indices were also assessed to identify conditions favoring biomass production and PUFA accumulation.

## 2. Materials and Methods

### 2.1. Elemental Analysis (CHNS) of Molasses and Other Components of the Culture Medium

The carbon source used in this research is beet molasses, which was obtained from the IANSA sugar industry, Temuco, Chile. It was used under the same conditions as when obtained from the plant (without any prior treatment). Molasses was prepared at different concentrations (10, 20, 30, 50, 70, and 100 g/L), sterilized by autoclaving (WTC Binder, Tuttlingen, Germany), and 35 mL of each concentration were subsequently lyophilized for elemental analysis. Elemental analysis was also performed on the other components of the culture medium (yeast extract and malt extract). For CHNS elemental analyses, 2 mg of the sample was mixed with 5 mg of catalyst (VO5) and placed into a tin cup. Then the sample was burned in a column at different temperatures according to the standard methods of the instrument (Thermo Scientific, FLASH 2000 CHNS elemental analyser, Waltham, MA, USA). The degassed samples were separated and the chromatograms were created. Finally, the mass percentage of each element was calculated [24].

### 2.2. Yeast, Culture Conditions and Inoculum Preparation

The yeast *Rhodotorula mucilaginosa* 6S (Accession GenBank PP209387.1.) was provided from the strain collection of the Microbiology laboratory of the Department of Veterinary Medicine, Faculty of Natural Resources of the Catholic University of Temuco. This strain was previously identified by sequencing in a previous study conducted by our research group [14]. The strain was maintained in Sabouraud agar at 4 °C, with subcultures being performed every three weeks. The preinoculum was prepared in 100 mL of sterilized liquid medium for 24 h at 150 rpm and 25 °C. This medium contained the following composition: glucose 20 g/L, ammonium sulfate 3.75 g/L, yeast extract 2 g/L, and malt extract 1 g/L. Subsequently, flasks containing 180 mL of medium were inoculated with 10% *v*/*v* of the preinoculum, under sterile conditions in a laminar flow hood. The experiments were performed in duplicate and incubated for 72 h at 150 rpm and 25 °C [6].

### 2.3. Experimental Design for the Production of Biomass and Lipids

To evaluate the effect of the interaction between the C/N ratio and temperature on biomass and total lipid production, a 2 × 6 full factorial design was implemented during the culture phase. Different molasses concentrations were used as the carbon source to obtain C/N ratios ranging from 4 to 14, while the nitrogen source concentration (ammonium sulfate, 3.75 g/L) and the concentrations of yeast and malt extracts were kept constant. The evaluated temperatures were 15 and 25 °C. A total of 24 experimental runs were performed in duplicate, without blocks or center points, using a second-order interaction (2FI) model to assess both the main effects and interactions between factors. In this design, the C/N ratio was considered a categorical factor with six levels, whereas temperature was treated as a numerical variable. The response variables analyzed included Biomass production (dry weight, g/L), Lipid content (%), Total lipids (g/L), Volumetric lipid productivity (mg/L·h), Specific growth rate (μ), Biomass/substrate yield (Y_X/S_), and Lipid/substrate yield (Y_P/S_) [25]. Supplementary Table S1 summarizes the experimental conditions evaluated.

### 2.4. Determination of Biomass Using Dry Weight Technique

For each experiment, 2 mL samples were taken from the flasks to measure biomass using the dry weight technique. The samples were filtered using filter paper (Advantec, GC-50, Tokyo, Japan) and a Kitasato flask (Duran, Schott, Mainz, Germany) connected to a vacuum generator motor. Subsequently, they were dried in an oven at 60 °C to achieve a constant weight. Biomass was determined by weight difference [26], and subsequently lyophilized [27].

### 2.5. Quantification of Total Lipids, Reducing Sugars and Total Sugars

Lipids were measured using the phosphoric vanillin lipid (PVL) assay. The yeast suspension (50 µL) was mixed with 1 mL of 18 M sulfuric acid in a threaded 13 × 100 mm Pyrex™ test tube (Corning # 9826-13, New York, NY, USA) and heated at 100 °C for 10 min in a dry heating bath. The solution was cooled for 5 min in an ambient water bath. Next, 2.5 mL of the vanillin- phosphoric acid was added and reacted for 15 min at 37 °C. The test tube was cooled for 10 min in water bath at ambient temperature. The absorbance of each reaction was read at 530 nm against a reference sample prepared with 50 µL water. Absorbance measurements were converted to lipid concentration using a calibration curve prepared with refined olive oil. Olive oil (100 mg) was dissolved in 20 mL of chloroform:methanol (2:1), and the stock solution was loaded into the assay mixture at a concentration of 50–250 mg/mL. The vanillin-phosphoric acid solution was prepared every day by dissolving 0.12 g vanillin in 20 mL of H_2_O and adjusting the volume to 100 mL with 85% o-phosphoric acid [28].

Reducing sugars were determined using the DNS methodology. Absorbance measurements were converted to reducing sugars concentration using a calibration curve prepared with glucose (0-2 g/L) [26]. The concentration of total sugars was determined by high-performance liquid chromatography (HPLC) (Alliance Waters e2695 Separation Module; Waters Inc, Milford, MA, USA). Samples were taken at the beginning (t = 0 h) and at the end of the experimental run (t = 72 h) for each C/N ratio and temperature evaluated. Concentrations of glucose, fructose, sucrose were measured. A sugar HPX-87H column (Bio-Rad Laboratories Inc., Hercules, CA, USA) held at 35 °C was used for measurements. The mobile phase was sulfuric acid (5 mM) at a flow rate of 0.6 mL min^−1^. A refractive index (Waters Inc., Milford, MA, USA) detector was used [29].

### 2.6. Growth Kinetics of Yeast

For growth kinetics, experiments were performed in duplicate in Erlenmeyer flasks with a volume of 200 mL of medium. Each flask was inoculated with an initial suspension of 10^6^ cells/mL of a 10% *v*/*v* yeast preculture in exponential phase. The flasks were incubated at 150 rpm for 72 h to different temperatures (15 and 25 °C). Samples of 2 mL were taken every 3 h to determine biomass, total lipids and reducing sugars. The growth kinetics and the kinetic parameters (specific growth rate (μ), maximum specific growth rate (μ_max_), substrate saturation constant (K_s_), biomass/substrate yield (Y_X/S_) and lipids/ substrate (Y_P/S_) were determined for the different experimental conditions [6].

### 2.7. Determination of Fatty Acid Profile by Gas Chromatography

To obtain the fatty acid methyl ester, the total lipids were methylated [30]. The methyl esters were injected (1 µL) using an autosampler connected to a chromatograph equipped with a flame ionization detector (FID), and helium (He) as carrier gas. The separation was carried out through a capillary column (Supelco, Bellefonte, PA, USA) (30 m length × 0.25 internal diameter × 0.20 µm film thickness). The injector and the detector temperatures were 220 and 200 °C, respectively. To ensure the best separation of fatty acids, the following programming was used: 60 °C for 1 min, followed by 4 °C per minute to 204 °C, and, finally, 2 °C per minute to 220 °C, and 2 min for stabilization. Fatty acids were identified by comparison with Supelco-37 standard fatty acids (Sigma-Aldrich, Darmstadt, Germany), quantified with HPCHEM Stations software B.02.01-SR1 [260] (Agilent Technologies, Santa Clara, CA, USA), and expressed as area percentage based on the total fatty acids identified [6].

#### Determination of Nutritional Indices

The nutritional indices of the lipids were calculated from the fatty acid composition obtained by gas chromatography with flame ionization detection (GC-FID) (Supplementary Tables S2 and S3). The relative percentages of each fatty acid were used to determine the different nutritional quality indices using the equations described in the literature [21,22,23]. The calculated indices were: DU (Degree of Unsaturation), AI (Atherogenic Index), TI (Thrombogenic Index), HH (Hypocholesterolemic/Hypercholesterolemic Ratio), DI (Desaturation Index), SI (Spreadability Index), and the omega-6/omega-3, LA/ALA, MUFA/SFA, and PUFA/SFA ratios. The equations used to calculate each index are:

(1) DU=MUFA+2∗(PUFA)

(2) $$AI=\frac{\left[C12:0+(4*C14:0)+C16:0\right]}{\sum UFA}$$

(3) $$TI=\frac{\left(C14:0+C16:0+C18:0\right)}{\left[(0.5*\sum MUFA)+(0.5*\sum n\text{-}6PUFAn)+(3*\sum n\text{-}3PUFA)+(n\text{-}3/n\text{-}6)\right]}$$

(4) $$HH=\frac{Ʃ(C18:1\omega \text{-}9+C18:1\omega \text{-}7+C18:2\omega \text{-}6+C18:3\omega \text{-}3+C18:3\omega \text{-}6+C20:3\omega \text{-}6+C20:4\omega \text{-}6+C20:5\omega \text{-}3+C22:5\omega \text{-}3+C22:6\omega \text{-}3)}{\left(C14:0+C16:0\right)}$$

(5) $$DI=\frac{C18:1\omega \text{-}9}{\left(C18:0+C18:1\omega \text{-}9\right)}$$

(6) $$SI=\frac{C18:1\omega \text{-}9}{C16:0}$$

(7) $$\frac{\omega -6}{\omega -3}=\frac{Ʃ\omega \text{-}6}{Ʃ\omega \text{-}3}$$

(8) $$\frac{LA}{ALA}=\frac{C18:2\;n\text{-}6}{C18:3\;n\text{-}3}$$

(9) $$\frac{MUFA}{SFA}=\frac{\sum MUFA}{\sum SFA}$$

(10) $$\frac{PUFA}{SFA}=\frac{\sum PUFA}{\sum SFA}$$

### 2.8. Statistical Analysis

A full factorial design was used for this research. In all cases, a 95% confidence level (α = 0.05) was considered. Analyses were performed using Design Expert 6.06 software. All culture techniques were performed in duplicate, while all response variables were measured in triplicate (Biomass (dry weight, g/L), Lipids (%), Total lipids (g/L), Volumetric lipid productivity (mg/Lh), Specific growth rate (μ), Biomass/substrate yield (Y_X/S_) and Lipids/substrate (Y_P/S_)). The results were subjected to analysis of variance (ANOVA). When significant differences were found between variables, with a significance level of 5% (*p* < 0.05), Tukey’s test for mean comparison was applied.

## 3. Results

### 3.1. CHNS Composition of Molasses and Culture Medium

Table 1 presents the elemental composition of the culture medium components, showing approximate values of 37% carbon (C) and 2% nitrogen (N) for molasses. In this study, different molasses concentrations (10–100 g/L) were evaluated as the carbon source, while the nitrogen source (ammonium sulfate, 3.75 g/L) and the concentrations of yeast and malt extracts were kept constant. These conditions resulted in different C/N ratios (4, 6, 8, 10, 12 and 14).

### 3.2. Effect of Variables on Biomass Production and Lipid Accumulation in R. mucilaginosa Cultured in Molasses

Table 2 summarizes the values obtained from the experimental design, including biomass, total lipids, lipid content (%), volumetric productivity, biomass/substrate yield (Y_x/s_), and lipid/substrate yield (Y_p/s_).

**Figure 1:**
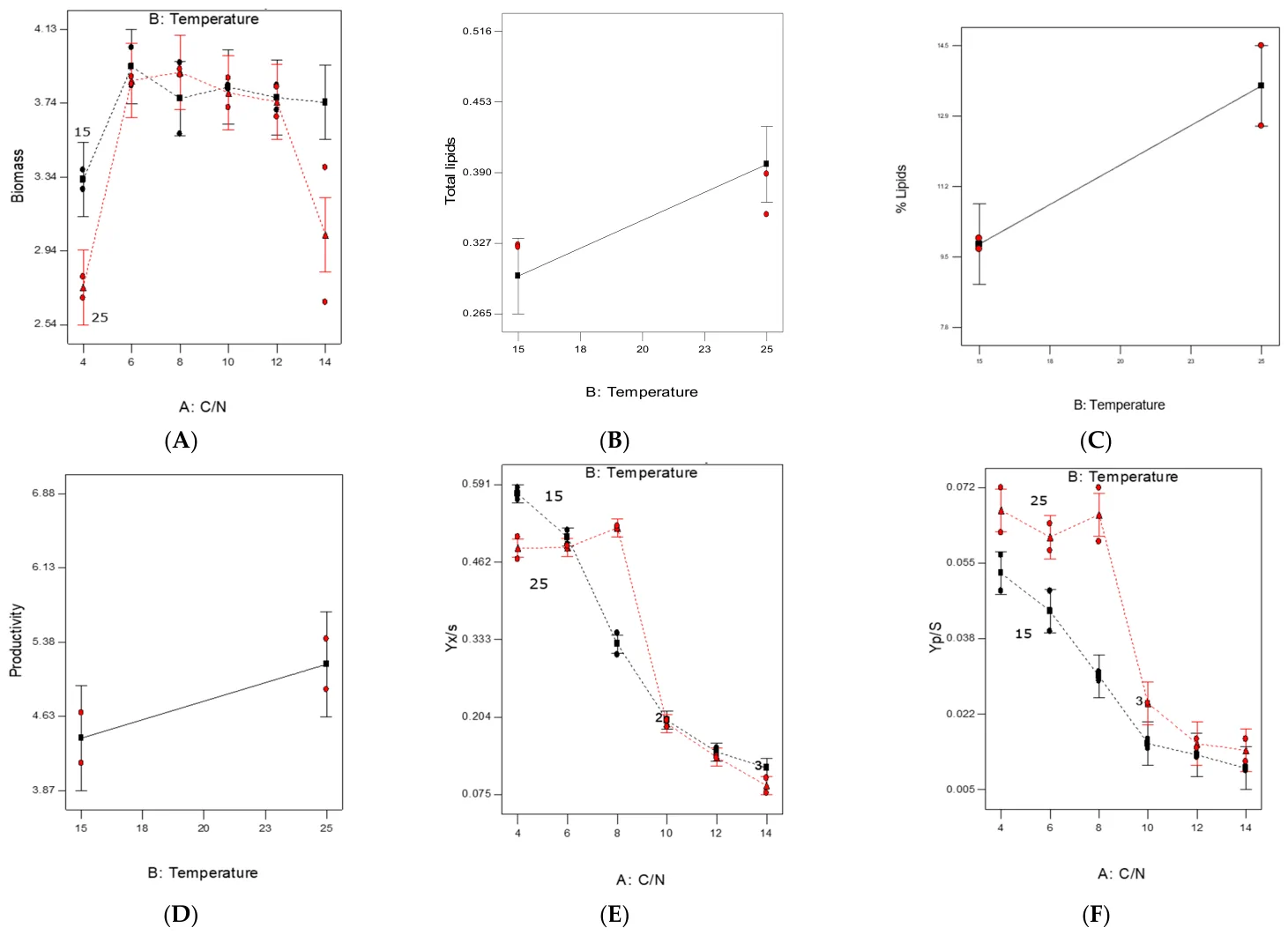
Effect of C/N ratio, temperature, and C/N × T interaction in response variables. (**A**) Interaction of the C/N ratio and temperature (°C) on biomass production (g/L). (**B**) Effect of temperature (°C) on total lipid production (g/L). (**C**) Effect of temperature (°C) on lipids (%). (**D**) Effect of temperature (°C) on volumetric productivity (mg/L·h). (**E**) Interaction of the C/N ratio and temperature (°C) on biomass/substrate yield (Y_X/S_). (**F**) Interaction of the C/N ratio and temperature (°C) on product/substrate yield (Y_P/S_). In the interaction graphs, the red dashed line corresponds to 25 °C, while the black dashed line corresponds to 15 °C. The dots represent design points, while the triangles represent a temperature of 25 °C and the squares a temperature of 15 °C.

Biomass production increased progressively in the flasks, ranging from 3.02 to 3.90 g/L at 25 °C and from 3.32 to 3.93 g/L at 15 °C at the different C/N ratios evaluated (4–14). The coefficient of determination (R^2^ = 0.886) demonstrated a good fit of the model, while the analysis of variance indicated that the model was highly significant (*p* = 0.0004), confirming that the C/N ratio, temperature, and their interaction adequately explained the observed variability in biomass production. The C/N ratio has a highly significant effect (*p* = 0.0001), being the factor that contributes most to the variation in biomass. This suggests that the carbon-nitrogen balance is crucial for cell growth, consistent with the metabolism of oilseed yeasts, where adequate nitrogen availability favors cell proliferation. Temperature also has a significant influence (*p* = 0.0148), indicating that thermal changes affect the growth rate. Furthermore, the C/N × T interaction is significant (*p* = 0.0337), demonstrating that the effect of the C/N ratio depends on the culture temperature. Figure 1A shows that as the C/N ratio increases, biomass values increase for both temperatures. However, this behavior changes at the C/N ratio (14) depending on the culture temperature. At 25 °C and a C/N ratio of 14, biomass decreases drastically, while at 15 °C and the same C/N ratio, biomass remains constant.

Total lipid production increased throughout the culture period, reaching 0.37–0.47 g/L at 25 °C and 0.30–0.35 g/L at 15 °C under the evaluated C/N ratios (4–14). The coefficient of determination (R^2^ = 0.768) demonstrated a good fit for the model. The analysis of variance shows that the model is significant (*p* = 0.0182), indicating that the C/N ratio, temperature, and their interaction adequately explain the observed variability in total lipid production. Temperature had a significant effect on total lipid production (*p* = 0.0001), with lipid production increasing from approximately 0.33 g/L at 15 °C to 0.42 g/L at 25 °C (Figure 1B). In contrast, the C/N ratio (*p* = 0.5158) and the C/N × T interaction (*p* = 0.5945) did not show a statistically significant effect, identifying temperature as the main factor influencing lipid production in this system.

Lipid production, expressed as a percentage, shows that the highest values were obtained at 25 °C (10.2–14.5%). The model for the percentage of lipids is highly significant (*p* = 0.0002) and has an R^2^ = 0.905, demonstrating a good explanation of the data variability. Temperature is the dominant factor (*p* < 0.0001) (Figure 1C), while the C/N ratio shows a non-significant effect (*p* = 0.0601). The interaction between these two factors is also not significant (*p* = 0.2862). These results indicate that temperature not only affects the absolute amount of lipids produced, but also their proportion relative to the total biomass, which is consistent with the behavior of oilseed yeasts, where specific thermal conditions favor the channeling of carbon towards lipid storage.

Consistent with lipid production (%), volumetric lipid productivity was higher at 25 °C (4.90–6.88 mg/Lh), and the model showed a significant fit (*p* = 0.0033; R^2^ = 0.834). Temperature was the only factor with a significant effect (*p* < 0.0001), whereas the C/N ratio (*p* = 0.2463) and its interaction with temperature (*p* = 0.4143) showed no statistical influence. Figure 1D indicates that volumetric productivity is mainly influenced by temperature, with higher productivity observed at 25 °C than at 15 °C, suggesting that increased temperatures favor yeast metabolic activity and lipid production.

The highest values of biomass/substrate yield (Y_X/S_) were obtained at 15 °C (0.576 and 0.503 g biomass/g substrate at C/N ratios of 4 and 6, respectively), indicating more efficient substrate conversion into biomass. In general, Y_X/S_ decreased as the C/N ratio increased, suggesting nitrogen limitation and a shift in metabolism toward maintenance or storage processes. The model was highly significant (*p* < 0.0001) and showed an excellent fit (R^2^ = 0.997). The C/N ratio and its interaction with temperature significantly affected Y_X/S_ (*p* < 0.0001), whereas temperature alone was not significant (*p* = 0.2384). Higher Y_X/S_ values were achieved at 15 °C across a broader C/N range, indicating greater biomass production efficiency (Figure 1E).

Product/substrate yield (Y_P/S_) also decreased with increasing C/N ratio at both temperatures. The highest yields (≈0.06–0.07 g lipids/g substrate) were obtained at low C/N ratios (4–8), particularly at 25 °C, indicating more efficient substrate conversion into lipids. At higher C/N ratios, Y_P/S_ declined markedly despite similar final lipid concentrations, suggesting carbon diversion to other metabolic processes. The model was highly significant (*p* < 0.0001) with an excellent fit (R^2^ = 0.980). Both temperature and C/N ratio significantly affected Y_P/S_ (*p* < 0.0001), and a significant interaction between these factors was observed (*p* = 0.0019), demonstrating that lipid production efficiency depends on their combined effect (Figure 1F). Overall, 15 °C favored substrate conversion into biomass, whereas low C/N ratios and 25 °C enhanced lipid production efficiency and provided the best overall performance for bioreactor scale-up.

### 3.3. Yeast Growth Profile

**Figure 2:**
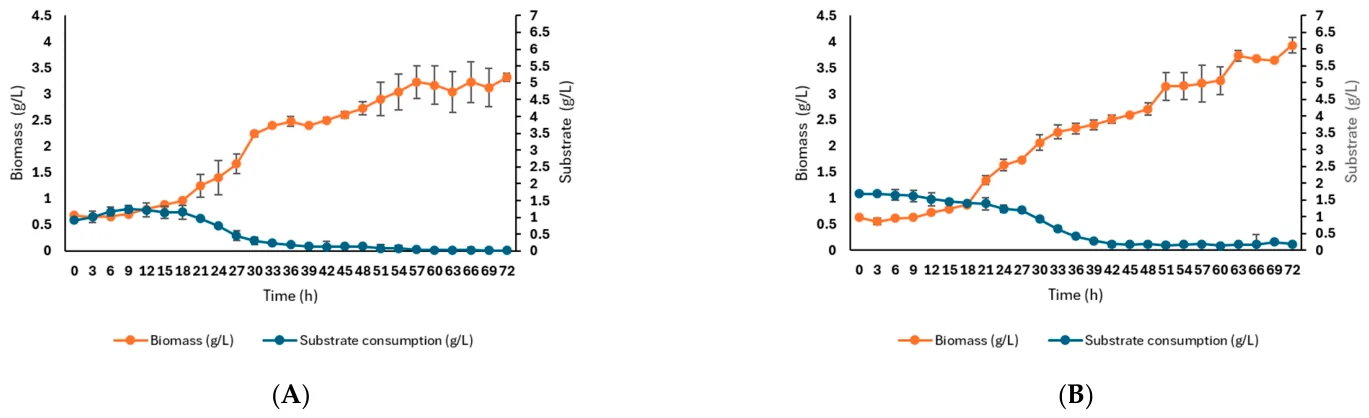

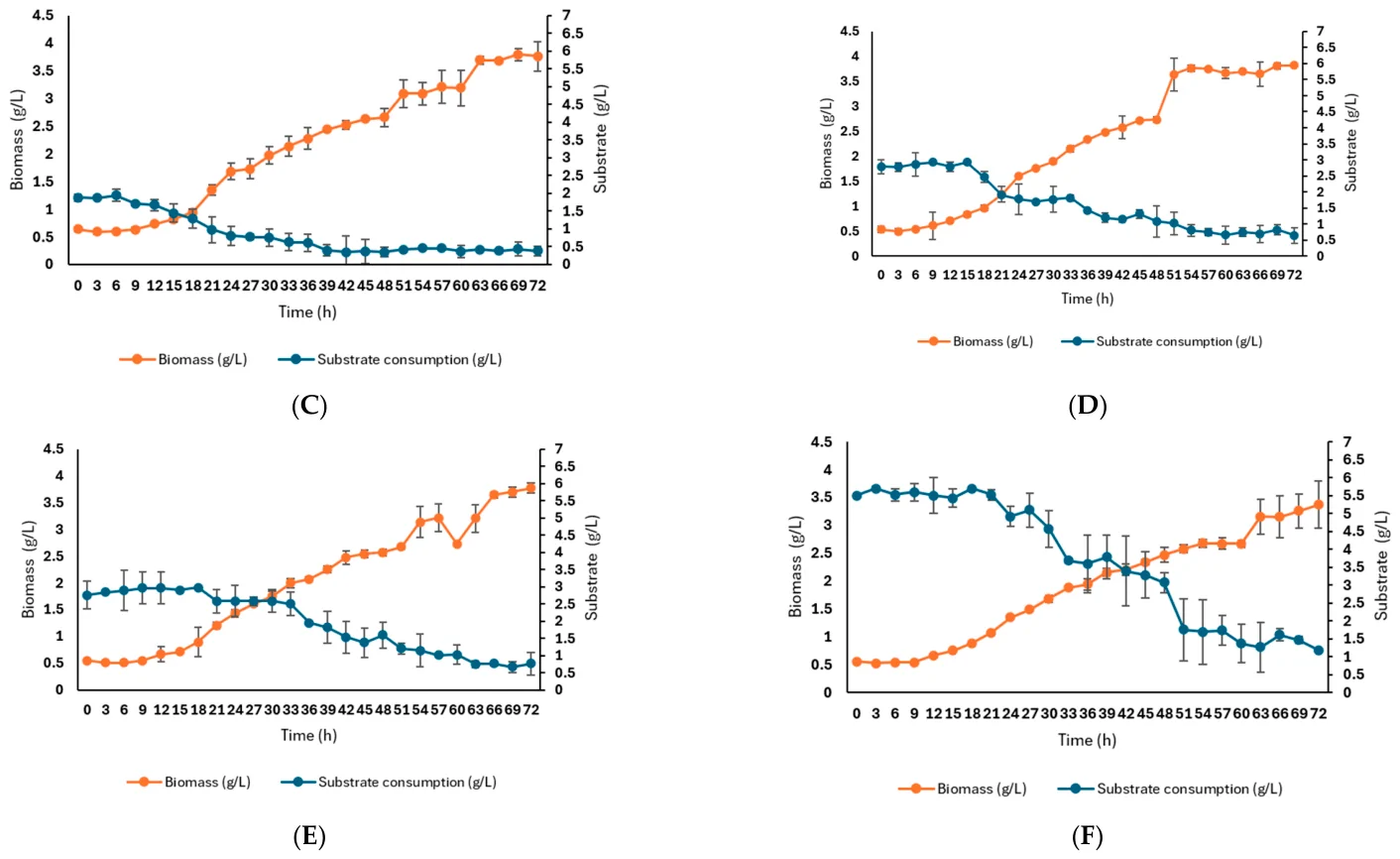
Growth kinetics (biomass production and substrate consumption) of *Rhodotorula mucilaginosa* 6S with different C/N ratios and a temperature of 15 °C. (**A**) C/N = 4; (**B**) C/N = 6; (**C**) C/N = 8; (**D**) C/N = 10; (**E**) C/N = 12; (**F**) C/N = 14. Substrate consumption was determined using the DNS methodology.

**Figure 3:**
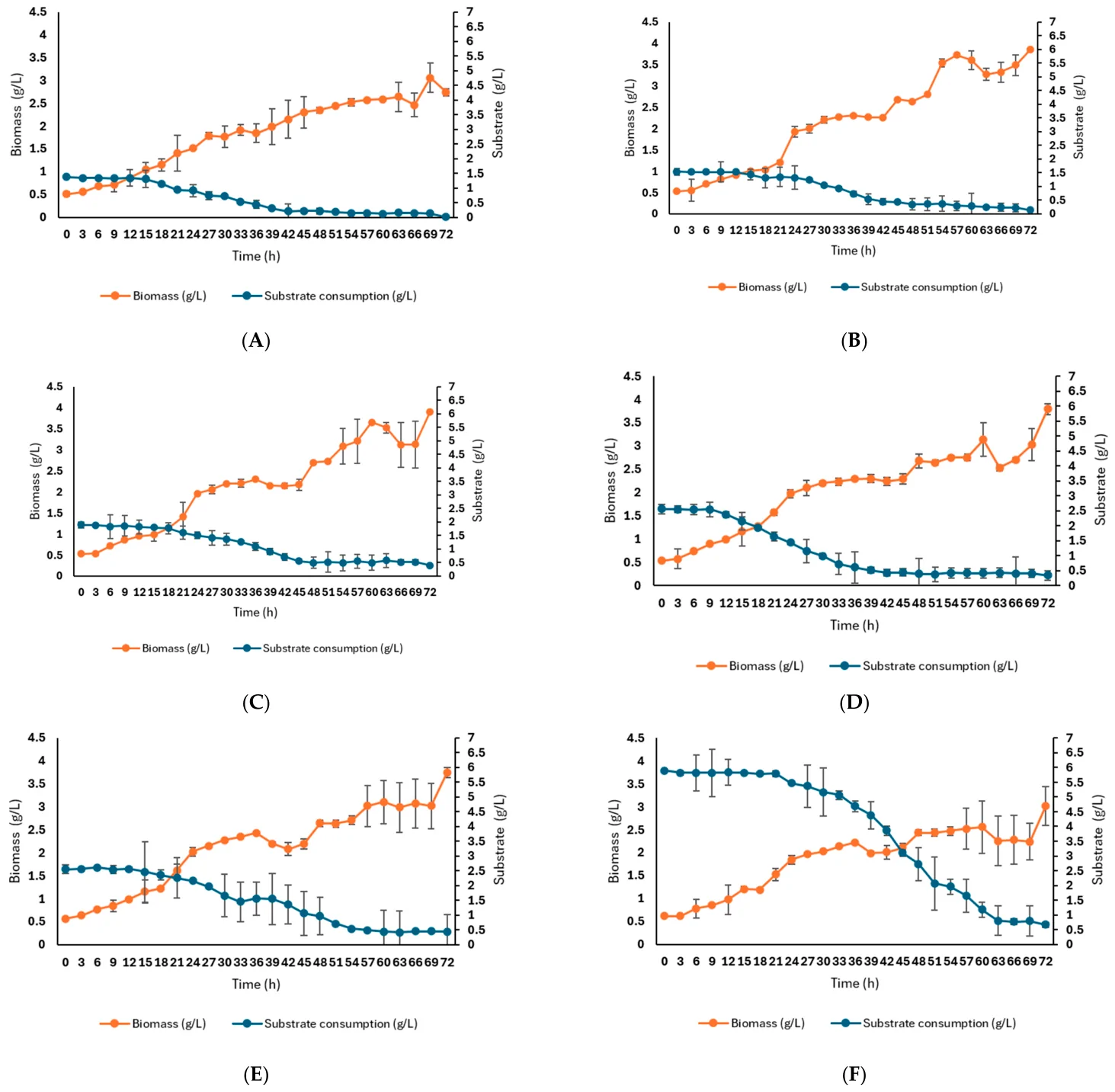
Growth kinetics (biomass production and substrate consumption) of *Rhodotorula mucilaginosa* 6S with different C/N ratios and a temperature of 25 °C. (**A**) C/N = 4; (**B**) C/N = 6; (**C**) C/N = 8; (**D**) C/N = 10; (**E**) C/N = 12; (**F**) C/N = 14. Substrate consumption was determined using the DNS methodology.

Figure 2 and Figure 3 show the growth kinetics (biomass production and substrate consumption) of *Rhodotorula mucilaginosa* 6S at the different C/N ratios evaluated (4–14) over time (72 h) at two temperatures (15 and 25 °C).

The growth curves of *Rhodotorula mucilaginosa* 6S showed the typical microbial growth pattern under all evaluated conditions, consisting of lag, exponential, and stationary phases, while no decline phase was observed during the 72 h cultivation period. The lag phase lasted approximately 6–9 h in all treatments, indicating that neither temperature nor the C/N ratio markedly affected the initial adaptation of the yeast to the culture medium.

Temperature mainly influenced the growth kinetics rather than the overall growth pattern. At 15 °C, cultures progressed more slowly and reached the stationary phase only after approximately 50 h, reflecting reduced metabolic activity and a slower rate of biomass accumulation. In contrast, cultures incubated at 25 °C entered the stationary phase earlier, indicating faster cell growth under this condition. Despite these differences in growth rate, no decline phase was observed, suggesting that the available nutrients were sufficient to maintain cell viability throughout the cultivation period.

The effect of temperature was also reflected in the optimal C/N ratio for biomass production. At 15 °C, the highest average biomass (3.93 g dry weight L^−1^) was obtained at a C/N ratio of 6, whereas at 25 °C the highest biomass (3.90 g dry weight L^−1^) was achieved at a C/N ratio of 8. Although the final biomass values were similar, cultures grown at 15 °C consumed more substrate, leaving a lower residual sugar concentration (0.19 g/L) than those grown at 25 °C (0.40 g/L). These results suggest that temperature influences substrate utilization and nutrient requirements, modifying the growth kinetics and the C/N ratio that maximizes biomass production without substantially affecting the final biomass yield.

**Figure 4:**
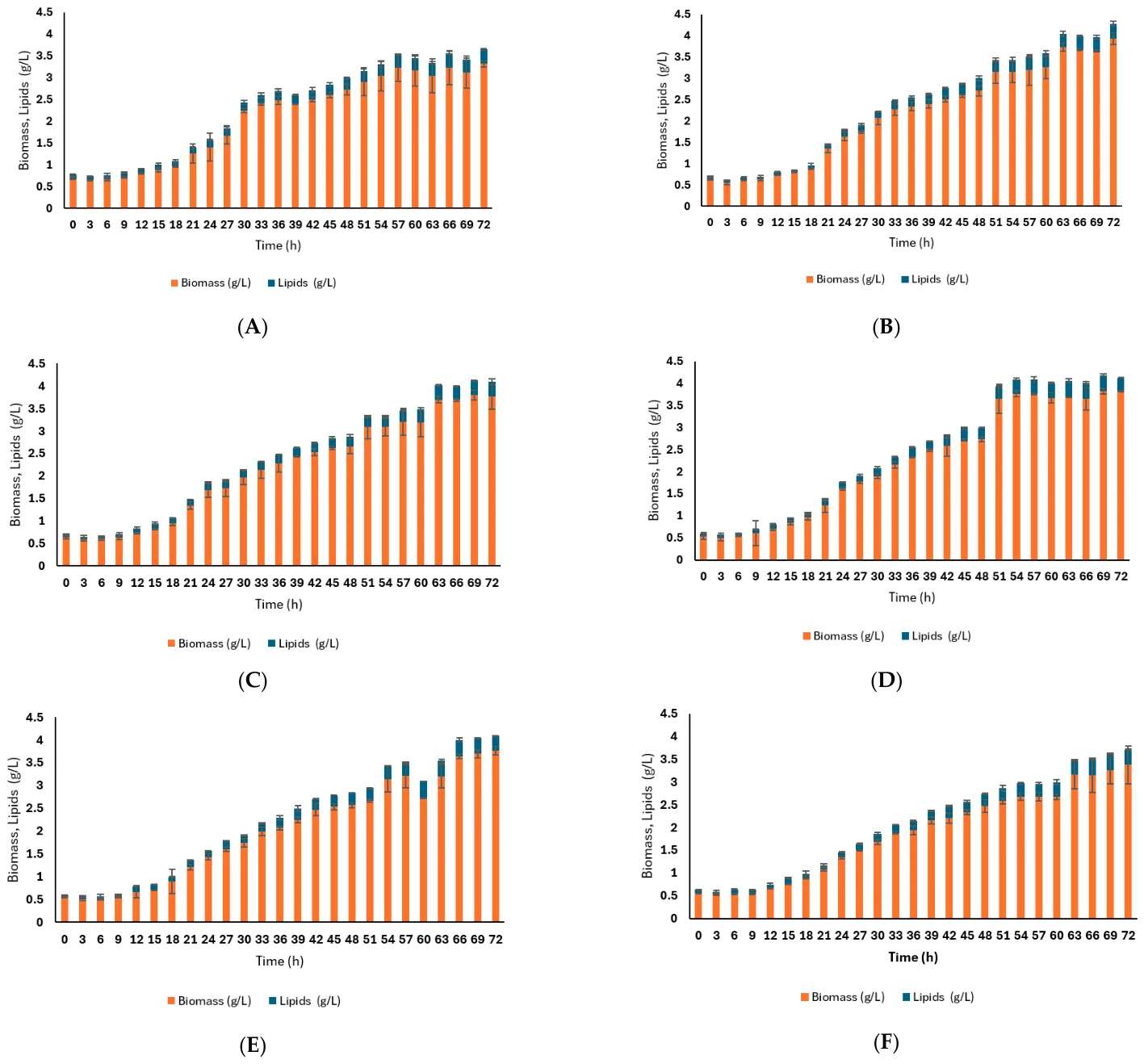
Growth kinetics (biomass and lipid production) of *Rhodotorula mucilaginosa* 6S with different C/N ratios and a temperature of 15 °C. (**A**) C/N = 4; (**B**) C/N = 6; (**C**) C/N = 8; (**D**) C/N = 10; (**E**) C/N = 12; (**F**) C/N = 14.

**Figure 5:**
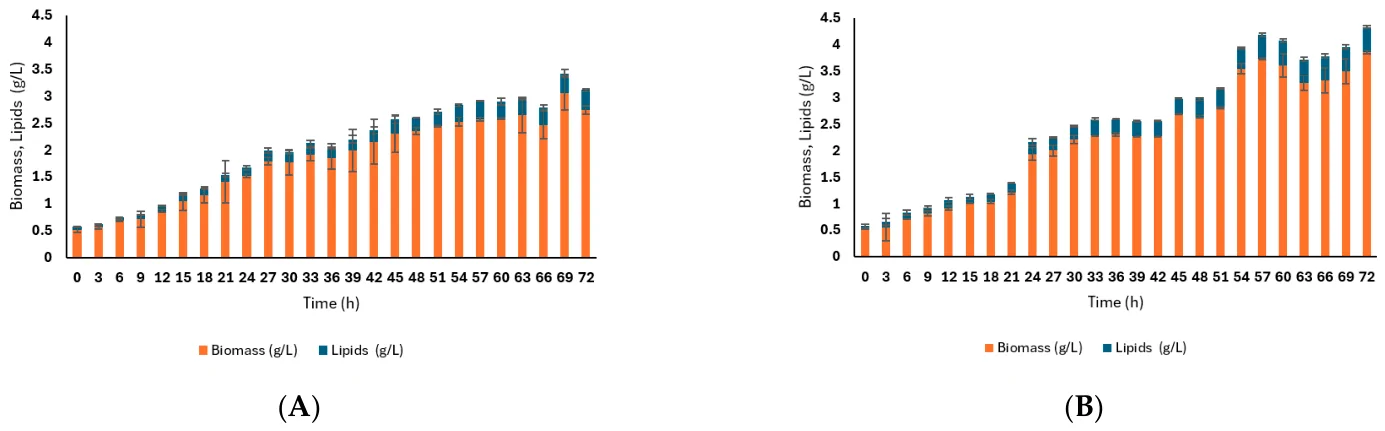

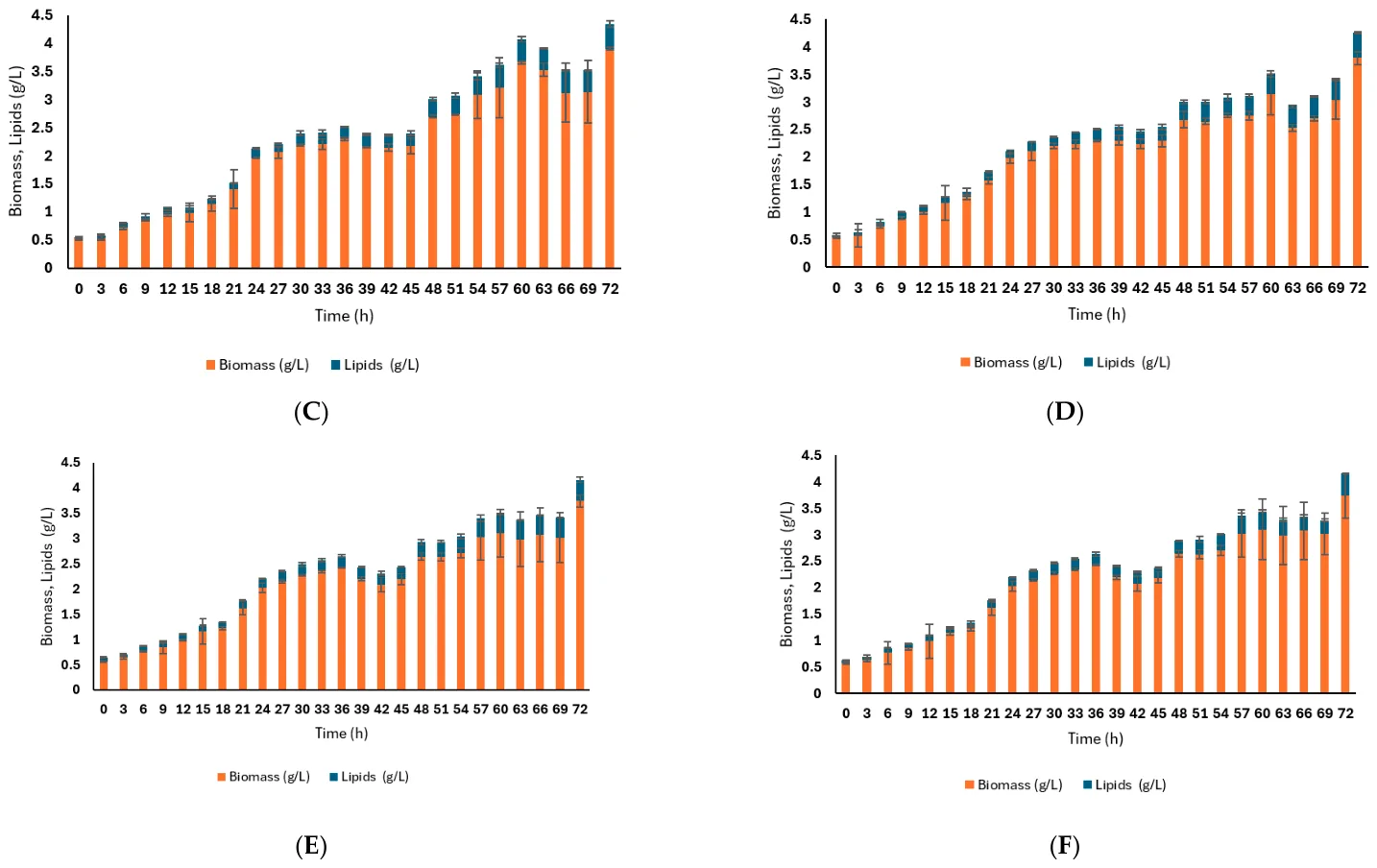
Growth kinetics (biomass and lipid production) of *Rhodotorula mucilaginosa* 6S with different C/N ratios and a temperature of 25 °C. (**A**) C/N = 4; (**B**) C/N = 6; (**C**) C/N = 8; (**D**) C/N = 10; (**E**) C/N = 12; (**F**) C/N = 14.

Figure 4 and Figure 5 show that the C/N ratio is a key factor in the growth kinetics and lipid accumulation of *Rhodotorula mucilaginosa* 6S, at both 15 °C and 25 °C. At both temperatures, low C/N ratios (4–6) primarily favor cell growth, while increasing the C/N ratio leads to a metabolic transition toward lipid accumulation, characteristic of oleaginous yeasts under nitrogen limitation. This behavior is evidenced by the progressive increase in lipid concentration in the intermediate and final stages of the culture, especially from C/N ≥ 8. Comparatively, at 25 °C, growth kinetics and lipid accumulation are faster and reach higher values than at 15 °C, indicating a positive effect of temperature on metabolic activity. However, under both thermal conditions, C/N ratios > 10 do not result in proportional increases in lipid production, suggesting the existence of an optimal C/N range (8–10) where the balance between biomass and lipids is maximized. This indicates that an excess of carbon, combined with an extreme nitrogen limitation, does not necessarily improve the productive efficiency of the system.

### 3.4. Kinetic Parameters

Supplementary Table S4 shows the combined effect of the C/N ratio and temperature (15 and 25 °C) on the specific growth rate (μ) under different experimental conditions.

To determine the specific growth rate (μ), the total sugar concentration in molasses was quantified by HPLC at the beginning (0 h) and end (72 h) of cultivation. Sucrose was the predominant sugar in all experimental conditions, followed by lower concentrations of glucose and fructose (Supplementary Table S5).

**Figure 6:**
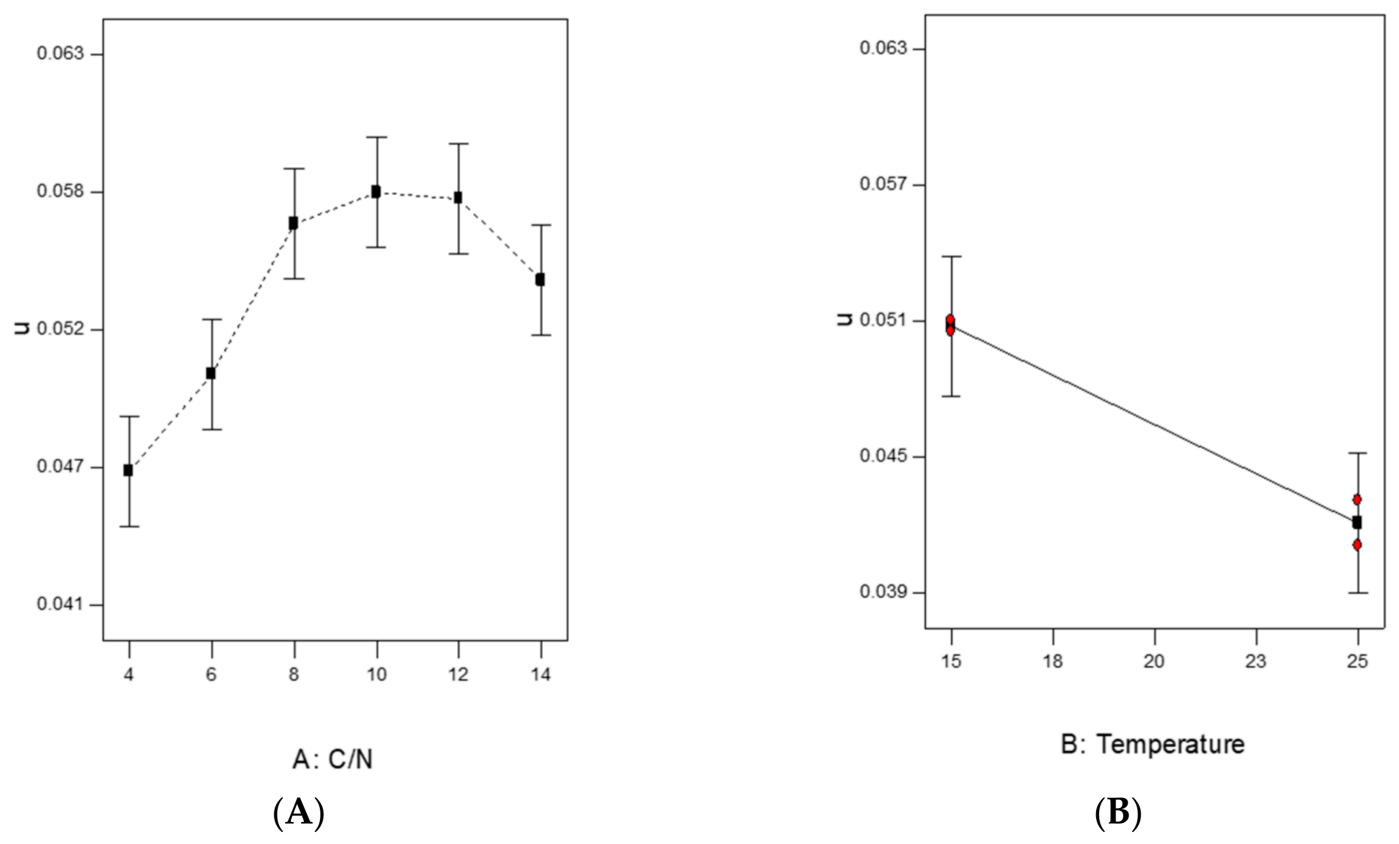
Effect of C/N ratio and temperature on specific growth rate (h^−1^). (**A**) Effect of C/N ratio. (**B**) Effect of temperature. The dots represent the design points.

The specific growth rates obtained for *Rhodotorula mucilaginosa* 6S were relatively low, which may be associated with the use of molasses as the carbon source. At 25 °C, μ ranged from 0.042 to 0.056 h^−1^, reaching its highest value at a C/N ratio of 14. A similar trend was observed at 15 °C, although slightly higher growth rates were recorded (0.051–0.059 h^−1^) (Supplementary Table S4). ANOVA revealed that the model was highly significant (*p* < 0.0001) and showed a good fit (R^2^ = 0.870). Both the C/N ratio (*p* = 0.0001) and temperature (*p* < 0.0001) significantly affected μ, whereas their interaction was not significant (*p* = 0.5301). Figure 6A shows that μ increased with the C/N ratio up to an optimum level, indicating that intermediate C/N ratios provide more favorable growth conditions, while extreme ratios limit microbial growth. Temperature also had a marked effect on growth, with higher μ values observed at 15 °C than at 25 °C (Figure 6B), suggesting that lower temperatures within the evaluated range favored the growth of *R. mucilaginosa* 6S.

The relationship between the inverse of the specific growth rate (1/μ) and the inverse of the substrate concentration (1/S) at 25 and 15 °C were fitted using linear regression. From these relationships the kinetic parameters were estimated, being μ_max_ = 0.058 h^−1^ and K_s_ = 1.717 g/L for 25 °C; while for 15 °C the values were μ_max_ = 0.062 h^−1^ and K_s_ = 0.946 g/L.

The μ_max_ values obtained confirm that temperature significantly influences microbial growth. At 15 °C, higher μ_max_ values and a lower K_s_ (0.946 g/L) were observed compared to 25 °C (1.717 g/L), indicating a greater affinity for the substrate and greater efficiency in its utilization under this thermal condition. This suggests that at lower temperatures, the microorganism maintains high growth rates even with limited substrate concentrations. In contrast, the higher K_s_ at 25 °C reflects a lower apparent affinity, possibly associated with higher energy requirements or metabolic stress. Overall, the combination of a high μ_max_ and a reduced K_s_ at 15 °C suggests that this temperature not only favors maximum growth but also substrate utilization efficiency. In processes where the substrate represents a significant cost or where moderate concentrations are used, operating at 15 °C would allow for efficient growth without the need for high carbon loads.

### 3.5. Fatty Acid Profile

**Figure 7:**
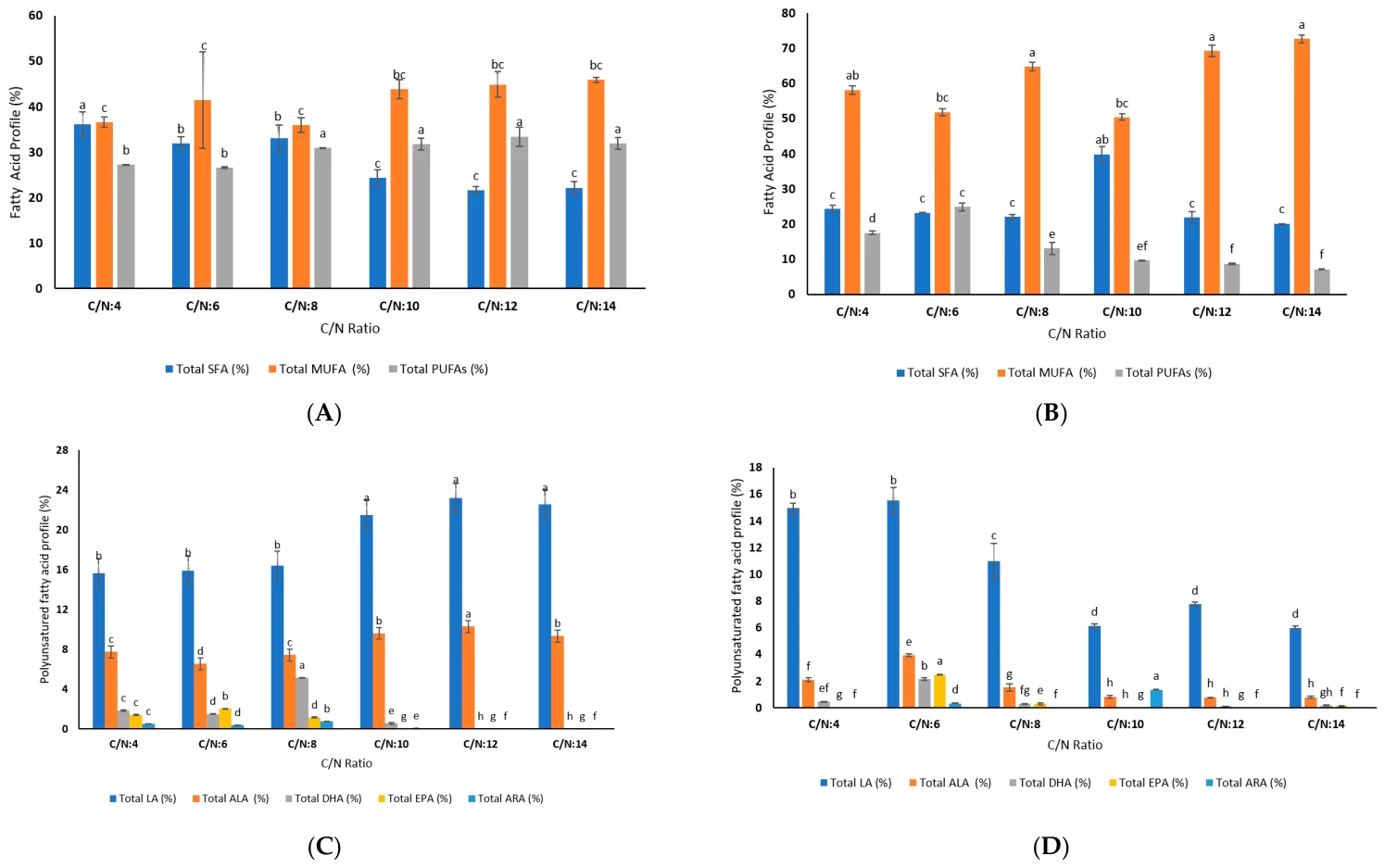
Lipid composition of *Rhodotorula mucilaginosa* 6S cultivated at different temperatures. (**A**,**C**) Fatty acid composition and (**B**,**D**) polyunsaturated fatty acid (PUFA) composition at 15 and 25 °C, respectively. SFA (Total Saturated Fatty Acids %), MUFA (Total Monounsaturated Fatty Acids %), PUFA (Total Polyunsaturated Fatty Acids %), LA (Linoleic Acid %), ALA (Linolenic Acid %), ARA (Arachidonic Acid %), EPA (Eicosapentaenoic Acid %) and DHA (Docosahexaenoic Acid %). (a, b, c, d, e, f, g) indicate significant differences between the variables through a Tukey test (*p* < 0.05): same letter, no significant difference, *p* < 0.05; different letters, statistical difference.

**Figure 8:**
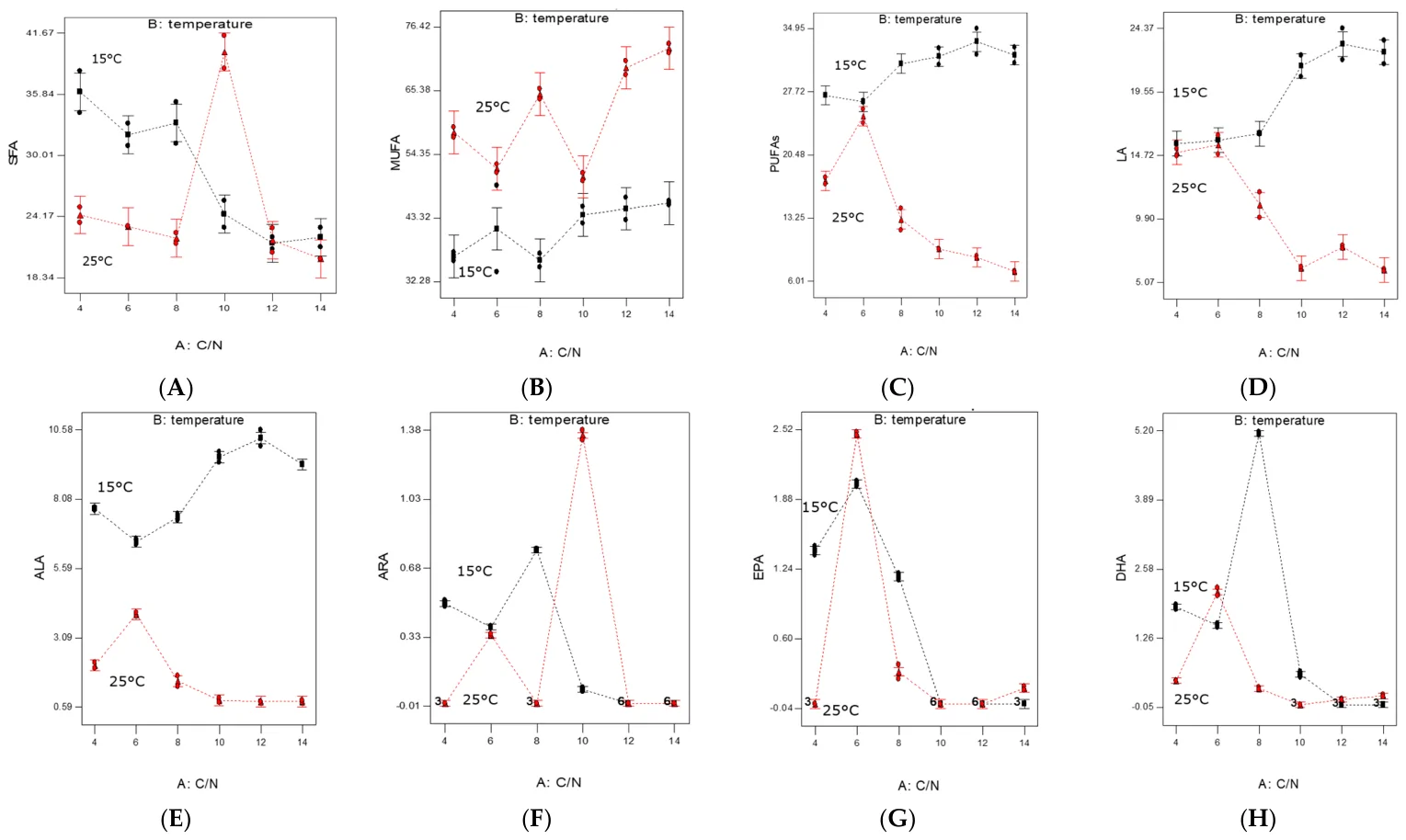
Effect of the C/N ratio and its interaction with temperature on fatty acid composition. (**A**) Total Saturated Fatty Acids (SFA) (%). (**B**) Total Monounsaturated Fatty Acids (MUFA) (%). (**C**) Total Polyunsaturated Fatty Acids (PUFAs) (%). (**D**) Linoleic Acid (LA) (%). (**E**) Linolenic Acid (ALA) (%). (**F**) Arachidonic Acid (ARA) (%). (**G**) Eicosapentaenoic Acid (EPA) (%). (**H**) Docosahexaenoic Acid (DHA) (%). In the interaction graphs, the red dashed line corresponds to 25 °C, while the black dashed line corresponds to 15 °C. The dots represent design points, while the triangles represent a temperature of 25 °C and the squares a temperature of 15 °C.

Figure 7 illustrates the variation in fatty acid composition and polyunsaturated fatty acid (PUFA) profile of lipids produced by *Rhodotorula mucilaginosa* 6S under different C/N ratios at cultivation temperatures of 15 °C and 25 °C. To further evaluate the combined influence of these cultivation factors, Figure 8 illustrates the interaction between temperature and the C/N ratio on the fatty acid composition, including total SFA, MUFA, PUFA (Figure 8A–C), and individual polyunsaturated fatty acids (LA, ALA, ARA, EPA and DHA) (Figure 8D–H).

The total saturated fatty acid (SFA) content was significantly influenced by the C/N ratio (*p* < 0.001), cultivation temperature (*p* = 0.0009), and their interaction (*p* < 0.001), indicating that lipid metabolism in *Rhodotorula mucilaginosa* is jointly regulated by nutritional and cultivation conditions (Figure 8A). Likewise, the monounsaturated fatty acid (MUFA) content was significantly affected by the C/N ratio (*p* = 0.0004), temperature (*p* < 0.0001), and their interaction (*p* = 0.0024), with temperature being the most influential factor, demonstrating that the response to changes in the C/N ratio depends on the cultivation temperature (Figure 8B). Similarly, the polyunsaturated fatty acid (PUFA) content was strongly influenced by the C/N ratio, temperature, and their interaction (*p* < 0.0001), with temperature again exerting the greatest influence (Figure 8C). Overall, these results demonstrate that variations in the C/N ratio and cultivation temperature jointly modulate the fatty acid profile of *R. mucilaginosa*, highlighting the importance of optimizing both factors to improve microbial lipids quality.

**Figure 9:**
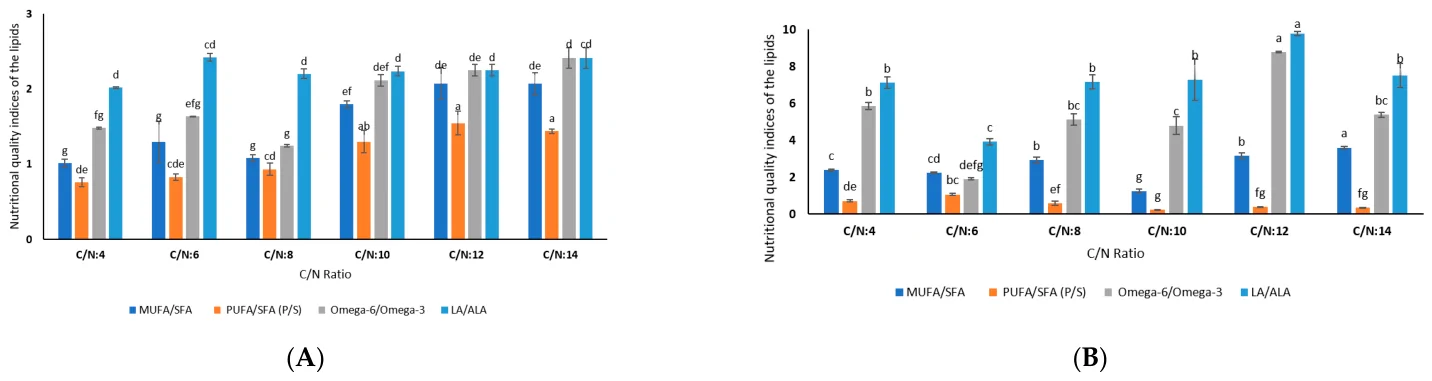
Nutritional quality of *Rhodotorula mucilaginosa* 6S cultivated at different temperatures. (**A**,**B**) Nutritional quality indices at 15 and 25 °C, respectively. (a, b, c, d, e, f, g) indicate significant differences between the variables through a Tukey test (*p* < 0.05): same letter, no significant difference, *p* < 0.05; different letters, statistical difference.

Within the PUFA fraction, the contents of linoleic acid (LA), α-linolenic acid (ALA), arachidonic acid (ARA), eicosapentaenoic acid (EPA), and docosahexaenoic acid (DHA) were differentially affected by the C/N ratio and cultivation temperature (Figure 7C,D and Figure 8D–H). LA content was significantly influenced by the C/N ratio (*p* = 0.0192), temperature, and their interaction (*p* < 0.0001), with temperature exerting the strongest effect, indicating that LA accumulation depends on the combined nutritional and thermal conditions. Similarly, ALA content was significantly affected by the C/N ratio, temperature, and their interaction (*p* < 0.0001), demonstrating that ALA synthesis is jointly regulated by both cultivation variables. In contrast, ARA production was primarily determined by the C/N ratio and its interaction with temperature (*p* < 0.0001), whereas temperature alone was not significant (*p* = 0.2958). The highest ARA content (~1.36%) was obtained at a C/N ratio of 10 and 25 °C, while cultivation at 15 °C shifted the optimum to a C/N ratio of 8 (~0.78%); moreover, ARA synthesis was completely suppressed at C/N ratios ≥ 12 regardless of temperature. EPA accumulation was also significantly influenced by the C/N ratio, temperature, and their interaction (*p* < 0.0001). The maximum EPA content (~2.5%) was achieved exclusively at a C/N ratio of 6 and 25 °C, whereas cultivation at 15 °C maintained a more stable but lower EPA production across the evaluated C/N ratios. Finally, DHA content was significantly affected by the C/N ratio, temperature, and their interaction (*p* < 0.0001), with the highest level (~5.15%) obtained at 15 °C and a C/N ratio of 8. Increasing the temperature to 25 °C markedly reduced DHA accumulation (maximum ~2.10% at C/N 6), and DHA production became nearly undetectable at C/N ratios above 8. Overall, these results demonstrate that variations in the C/N ratio and cultivation temperature strongly modulate the PUFA composition of *Rhodotorula mucilaginosa* 6S, with each fatty acid exhibiting a distinct optimal combination of cultivation conditions. These changes in fatty acid composition were directly reflected in the nutritional quality of the microbial lipids, as evidenced by the calculated nutritional indices (Figure 9).

The nutritional indices of the lipids produced by *Rhodotorula mucilaginosa* 6S showed a clear effect of temperature and the C/N ratio on the quality of the lipid fraction (Figure 9). In general, at 15 °C, the values were more stable and balanced across the different C/N ratios, while at 25 °C, greater variability was observed, especially in the omega-6/omega-3 and LA/ALA ratios. At 15 °C, the MUFA/SFA and PUFA/SFA ratios tended to increase with the C/N ratio, particularly between C/N 10 and C/N 14, indicating a higher relative proportion of unsaturated fatty acids compared to saturated fatty acids. At this temperature, the omega-6/omega-3 and LA/ALA ratios remained within moderate ranges, suggesting a more nutritionally balanced lipid profile. At 25 °C, the omega-6/omega-3 and LA/ALA ratios increased markedly, especially at C/N 12 and C/N 14. This behavior indicates a greater relative accumulation of linoleic acid compared to α-linolenic acid, favoring a profile more oriented towards omega-6 fatty acids. However, this increase does not necessarily imply better overall nutritional quality, since a more moderate balance between omega-6 and omega-3 is generally considered more favorable. To further assess the nutritional and functional quality of the lipids produced under the different cultivation conditions, additional quality indices related to the degree of unsaturation and the potential health benefits of the fatty acid profile were evaluated (Table 3).

The lipid quality indices of the lipids produced by *Rhodotorula mucilaginosa* 6S showed a marked influence of temperature and C/N ratio on the degree of unsaturation and the nutritional potential of the produced lipids. Overall, DU values were high at both temperatures, indicating a relevant presence of unsaturated fatty acids. At 15 °C, DU progressively increased from C/N 4 to C/N 12, reaching its maximum value at C/N 12, suggesting that lower-temperature conditions favored greater lipid unsaturation. This behavior is consistent with the adaptive response of yeasts to low temperatures, in which an increase in unsaturated fatty acids contributes to maintaining cell membrane fluidity. From a nutritional perspective, AI and TI values were low under most of the evaluated conditions, indicating a potentially favorable lipid profile of the yeast lipids, since this fatty acid composition is associated with lower atherogenic and thrombogenic potential. At 15 °C, AI and TI progressively decreased as the C/N ratio increased, reaching the lowest values at C/N 12 and C/N 14. In particular, the treatment at 15 °C with a C/N ratio of 12 showed one of the most favorable profiles, combining high DU, low AI, low TI, and a high HH index. The HH index, which reflects the ratio between hypocholesterolemic and hypercholesterolemic fatty acids, increased with the C/N ratio, especially at 15 °C, reaching values close to 4.0 at C/N 12 and C/N 14. This indicates a higher relative proportion of fatty acids considered beneficial from a cardiovascular perspective. At 25 °C, HH values were also high, particularly at C/N 12 and C/N 14, where the highest values at this temperature were observed. However, at 25 °C and C/N 10, a distinct and atypical lipid profile was observed, characterized by the highest AI and TI values and the lowest HH value among the evaluated conditions. This pattern was associated with a higher proportion of saturated fatty acids, the detection of C18:1n9t and trans-C18:2 isomers, and a marked reduction in cis-oleic acid. Because trans fatty acids are uncommon components of oleaginous yeast lipids, their occurrence should be interpreted cautiously. Proximate characterization of the molasses confirmed the presence of a residual lipid fraction (0.08% crude fat), corresponding to approximately 40 mg L^−1^ of exogenous lipid in the culture medium at the 50 g L^−1^ molasses level. Since yeasts cultivated in molasses can incorporate substrate-derived fatty acids into cellular lipids, the detected trans isomers may partly reflect ex novo uptake and subsequent cellular modification of lipid components derived from the substrate, rather than exclusively de novo biosynthesis. Nevertheless, because the specific fatty acid composition of the molasses lipid fraction was not determined, the precise origin of these trans isomers cannot be established from the present experimental design. The DI remained relatively high and stable in most treatments, with values ranging from 0.80 to 0.96, indicating relevant desaturation activity in the yeast lipids. The highest values were mainly observed at 25 °C, although without major variations among C/N ratios, suggesting that desaturation was active at both temperatures but modulated by the relative availability of carbon and nitrogen.

On the other hand, SI showed a tendency to increase with the C/N ratio, especially at 25 °C, where it reached its maximum value at C/N 14. This result suggests that conditions with greater carbon availability favored the accumulation of lipids with a higher oiliness index. Nevertheless, when all indicators are considered together, cultivation at 15 °C with C/N ratios between 10 and 14, particularly C/N 12, appears to offer the best balance between degree of unsaturation, low atherogenic/thrombogenic potential, and high HH ratio.

## 4. Discussion

*Rhodotorula* spp. is an oleaginous yeast recognized for its ability to produce lipids and carotenoids from a wide variety of carbon sources [9], and its biomass production, lipid accumulation, and fatty acid composition are strongly influenced by factors such as the carbon source, the carbon-to-nitrogen (C/N) ratio, and temperature [5]. Owing to its high lipid productivity and fatty acid profile similar to vegetable oils, *Rhodotorula* has emerged as a promising microorganism for the sustainable production of microbial lipids for food and industrial applications [10]. In this context, molasses represents an attractive carbon source due to its high sugar content and low nitrogen concentration [20], as confirmed by the elemental analysis performed in this study, supporting its suitability as a substrate for lipid biosynthesis. Therefore, this study evaluated the combined effect of molasses concentration and temperature on biomass and lipid production by *Rhodotorula mucilaginosa* 6S. Molasses was used to establish C/N ratios between 4 and 14, and cultures were grown at 15 and 25 °C to determine the conditions that favor microbial growth and lipid accumulation.

Previous studies have shown that the optimal C/N ratio for biomass and lipid production varies considerably among oleaginous yeasts. Moderate C/N ratios have been reported to support growth and lipid accumulation in several species, including *Lipomyces starkeyi* at a C/N ratio of 17.9 [31]; *Saccharomyces cerevisiae* at C/N ratios of 7–14, with the highest glucose-6-phosphate dehydrogenase activity observed at a C/N ratio of 10 [32]; and *Rhodotorula mucilaginosa*, which exhibited optimal lipid accumulation (~20%) at a C/N ratio of 12.5 [33]. In contrast, other studies have reported high biomass concentrations and lipid contents at substantially higher C/N ratios, such as 50 and 80, in native oleaginous yeasts cultivated in residual vinasse [6]. The C/N ratios evaluated in the present study (4–14) therefore fall within the range of values reported in the literature for oleaginous yeast cultivation [34].

The maximum biomass/substrate yield (Y_X/S_) value obtained in this study (0.576 g biomass/g substrate) was within the range reported for *Rhodotorula mucilaginosa* cultivated in batch bioreactors (0.50 g/g) and higher than those obtained in molasses-based media for *Rhodotorula* spp. and *Rhodosporidium toruloides*, where values between 0.226 and 0.512 g/g have been described [20,35,36]. These results indicate that the low C/N ratios evaluated in the present study promoted efficient substrate utilization for biomass production, even when molasses was used as the sole carbon source. Furthermore, the results for *Rhodotorula mucilaginosa* 6S revealed that biomass production and Y_X/S_ were significantly influenced by both the C/N ratio and temperature, confirming the key role of these cultivation parameters in yeast growth performance. The highest biomass concentrations and Y_X/S_ values were obtained at low C/N ratios and 15 °C, indicating that nitrogen availability favored protein synthesis, cell proliferation, and efficient substrate conversion into biomass. The progressive decrease in Y_X/S_ with increasing C/N ratio suggests the onset of nitrogen limitation, which reduces biomass formation efficiency as excess carbon becomes available. This behavior is consistent with previous observations showing that nitrogen-rich conditions promote cell growth, whereas increasing carbon excess shifts metabolism away from biomass production [37]. Temperature also affected biomass formation, with the highest biomass values observed at 15 °C. This response differs from that reported for *Rhodotorula mucilaginosa* and *Lodderomyces elongisporus*, in which biomass production increased with increasing incubation temperature [38]. However, the present results are consistent with evidence indicating that the effect of temperature on biomass accumulation is highly strain-dependent. For instance, *Rhodotorula glacialis* showed no significant differences in biomass production across the temperatures evaluated, although its growth rate was affected [39]. Similarly, *R. mucilaginosa* has been reported to achieve a maximum biomass concentration of 21.5 ± 2.4 mg/mL after 15 days of incubation at 7 °C [40]. These findings, together with the higher biomass production observed at 15 °C in the present study, suggest that certain *Rhodotorula* strains may retain substantial biomass-forming capacity under relatively low-temperature conditions, highlighting the importance of strain-specific responses to temperature.

The higher biomass observed at 15 °C may be associated with the psychrotolerant characteristics of the studied strain, which could be related to its environmental origin, as *R. mucilaginosa* 6S was isolated from soil during a cold period. Although this does not demonstrate physiological adaptation, the environmental conditions from which the strain was isolated may have selected for traits that favor growth under relatively low-temperature conditions. Such adaptation may involve coordinated mechanisms associated with membrane homeostasis, cryoprotection, and the maintenance of cellular metabolic activity. In particular, an increased proportion of unsaturated fatty acids, including polyunsaturated fatty acids (PUFAs), can help preserve membrane fluidity and facilitate nutrient transport and cellular functions under cold conditions. Likewise, the accumulation of compatible solutes such as trehalose may contribute to the stabilization of cellular membranes and proteins during cold stress [41,42]. In addition to these mechanisms, psychrotolerant *R. mucilaginosa* has also been reported to produce cold-active enzymes that retain high catalytic activity at low temperatures. In particular, a cold-active carboxymethyl cellulase (CMCase) with an optimum activity around 20 °C and broad activity over low temperatures has been proposed to sustain metabolic processes under cold conditions, while the occurrence of two temperature optima for enzyme activity has been suggested as a mechanism contributing to psychrotolerance [43]. Therefore, the enhanced biomass accumulation observed at 15 °C in strain 6S may reflect the combined contribution of mechanisms associated with membrane homeostasis, cryoprotection, and the maintenance of enzymatic activity under relatively low-temperature conditions, providing a plausible physiological explanation for the strain-dependent response observed in this study.

Lipid production exhibited a different response pattern compared to biomass formation. Although lipid accumulation in oleaginous yeasts is generally associated with severe nitrogen limitation and high C/N ratios [44], significant lipid yields were obtained in the present study even within the relatively low C/N range evaluated (4–14). These results are particularly relevant because they demonstrate substantial lipid accumulation under nitrogen-rich conditions, supporting previous reports that some *Rhodotorula* strains are capable of producing lipids efficiently without requiring extreme carbon-to-nitrogen ratios. A possible explanation is that, under nitrogen-rich conditions, AMP deaminase activity may be inhibited, preventing the metabolic changes that typically redirect citrate through the citrate–malate translocase (CMT) pathway toward lipid biosynthesis [31]. However, the literature presents contrasting evidence among oleaginous yeasts. For example, *Lipomyces starkeyi* and *Trichosporon fermentans* have shown substantially lower lipid accumulation at low C/N ratios [45,46]. Furthermore, C/N ratios below 12 have traditionally been associated with carbon-limited growth, in which cells prioritize protein synthesis and maintain relatively low levels of carbohydrate and lipid reserves [37]. Considering that most experimental conditions evaluated in this study were within a low C/N range (4–14), it is likely that the cells did not experience a sufficiently strong nitrogen limitation to activate a distinct lipogenic metabolic state.

Despite these physiological constraints, the Y_P/S_ values obtained in the present study (0.06–0.07 g lipids/g substrate) were within the range reported for other oleaginous yeasts. For example, *Rhodotorula mucilaginosa* CCT3892 achieved lipid yields of 0.079 ± 0.005 and 0.038 ± 0.006 g lipids/g substrate when cultivated on glucose and hydrolyzed residual molasses, respectively, whereas supplementation with yeast extract reduced the yield to 0.010 ± 0.002 g lipids/g substrate due to preferential biomass formation [36]. Likewise, *Rhodotorula mucilaginosa* SMY40 cultivated with 70 g/L glucose reached a yield of 0.025 g lipids/g substrate [47], while *Rhodotorula glutinis* grown on sugarcane molasses achieved a higher yield of 0.21 g lipids/g total reducing sugars [48]. Overall, these comparisons indicate that the lipid yields obtained in this study are comparable to those reported for *Rhodotorula* species cultivated under a wide range of nutritional and operational conditions.

Consequently, the observed differences in lipid production were likely driven primarily by temperature. The highest Y_P/S_ values were obtained at low C/N ratios and 25 °C, indicating that temperature played a major role in regulating lipid synthesis under the experimental conditions evaluated. The significant influence of temperature on lipid production is consistent with previous studies demonstrating that thermal conditions strongly affect lipid biosynthesis, fatty acid composition, and lipid accumulation in oleaginous yeasts [6,38]. In addition, temperature was the main factor affecting volumetric lipid productivity, with 25 °C resulting in the highest productivity values and overall process performance. Similar findings have been reported for *Metschnikowia pulcherrima* and *Rhodotorula glutinis*, where cultivation temperatures between 20 and 25 °C promoted the highest lipid production rates [49]. However, the influence of temperature on lipid accumulation is not universal among oleaginous yeasts. For example, lipid accumulation in *Rhodotorula glacialis* has been reported to remain relatively stable across a broad temperature range [39].

Besides affecting lipid productivity, temperature also influenced the fatty acid profile of the lipids produced. Previous studies have shown that *Rhodotorula mucilaginosa* and *Lodderomyces elongisporus* cultivated at 15 °C accumulated high lipid contents (48–54%) and exhibited a greater degree of fatty acid unsaturation than cultures grown at higher temperatures [38]. Similarly, gas chromatography analysis in the present study showed that the highest proportion of polyunsaturated fatty acids was obtained at 15 °C. This finding suggests that lower cultivation temperatures favor the synthesis of unsaturated fatty acids, thereby modifying lipid composition and enhancing the nutritional quality of the microbial lipids [38].

The kinetic parameters obtained in this study were within the range reported for *Rhodotorula* species cultivated on molasses and other agro-industrial substrates, although the specific growth rates were generally lower than those reported under optimized conditions [6,20]. At 25 °C, the specific growth rate increased from 0.042 h^−1^ at a C/N ratio of 4 to a maximum of 0.056 h^−1^ at a C/N ratio of 14, while slightly higher values (0.051–0.059 h^−1^) were observed at 15 °C. These values were lower than those reported for oleaginous yeasts cultivated in vinasse, including 0.1388 h^−1^ for *Debaryomyces* sp., 0.11 h^−1^ for *Apiotrichum dulcitum*, and 0.19 h^−1^ for *Rhodotorula glutinis* [6]. However, the maximum specific growth rates (µ_max_) obtained in the present study (0.058 h^−1^ and 0.062 h^−1^, to 25 °C and 15 °C, respectively) were comparable to those reported for *R. toruloides* and *R. glutinis* cultivated in molasses-based media, where values of 0.091 and 0.065 h^−1^ were observed [20]. In contrast, these values were considerably lower than the µ_max_ of 0.144 h^−1^ previously reported for the same *Rhodotorula mucilaginosa* 6S strain under control conditions without selenium supplementation, highlighting the strong influence of cultivation conditions on yeast growth performance [14].

However, growth performance alone does not determine the biotechnological value of microbial lipids, making it essential to evaluate the nutritional quality of the lipids accumulated under different cultivation conditions. In this context, the fatty acid composition and nutritional quality indices confirmed that temperature and the C/N ratio were the main factors modulating the lipid profile of *R. mucilaginosa* 6S. Overall, both cultivation temperatures promoted lipids rich in unsaturated fatty acids, although distinct response patterns were observed. The degree of unsaturation (DU) ranged from 69.84 ± 0.87 to 111.84 ± 6.96, reaching its highest value at 15 °C and C/N 12, indicating that low-temperature cultivation favored a more unsaturated lipid profile [50]. This response is consistent with the adaptive strategy of oleaginous yeasts to maintain membrane fluidity under cold conditions through increased synthesis of unsaturated fatty acids [10,51]. This finding is particularly relevant because the nutritional quality of a lipid source depends not only on its total lipid content, but also on the relative proportions of saturated (SFA), monounsaturated (MUFA), and polyunsaturated fatty acids (PUFA) [52,53,54]. Accordingly, the MUFA/SFA and PUFA/SFA ratios further highlighted the effect of cultivation temperature on lipid quality. At 15 °C, both indices increased with the C/N ratio, reaching their highest values at C/N 12–14, which reflects a more balanced proportion of unsaturated relative to saturated fatty acids. In contrast, although cultivation at 25 °C promoted relatively high MUFA/SFA values, particularly at C/N 12 and C/N 14, the PUFA/SFA ratio declined under these conditions. These results indicate that low-temperature cultivation favored a lipid profile with a greater contribution of polyunsaturated fatty acids, which is generally considered nutritionally more desirable.

The effect of cultivation temperature on the nutritional balance of the produced lipids was further evidenced by the ω-6/ω-3 and LA/ALA ratios. At 15 °C, these ratios remained within moderate ranges, with ω-6/ω-3 values between 1.247 and 2.414 and LA/ALA values between 2.018 and 2.414. In contrast, at 25 °C, both ratios increased markedly, especially at C/N 12, where ω-6/ω-3 ratio reached 8.769 ± 0.04 and LA/ALA reached 9.775 ± 0.11. This pattern indicates a greater relative accumulation of linoleic acid compared with α-linolenic acid under higher-temperature conditions. From a nutritional perspective, these findings highlight that the quality of a lipid source depends not only on its total unsaturated fatty acid content but also on the balance between the omega-6 and omega-3 fatty acid families.

The desaturation index (DI) remained consistently high (0.80–0.96) across all cultivation conditions, indicating active fatty acid desaturation regardless of temperature. The highest DI values were observed at 25 °C with C/N ratios of 4, 8, 12, and 14, as well as at 15 °C with a C/N ratio of 12, suggesting that the yeast maintained an efficient desaturation capacity while the final fatty acid composition was primarily determined by the interaction between temperature and C/N ratio. This response is consistent with the importance of unsaturated fatty acids in membrane structure, cell signaling, and metabolic regulation [55,56], and agrees with previous observations in *R. mucilaginosa* CCT 7688 [57].

The oiliness index (SI) ranged from 1.20 to 4.60 and generally increased with the C/N ratio, particularly at 25 °C, where the highest value was recorded at C/N 14. This trend suggests that carbon-rich conditions promoted the accumulation of lipids with a higher oiliness index. However, SI alone did not reflect the overall nutritional quality of the oil, indicating that it should be interpreted together with complementary indices describing fatty acid composition and health-related properties.

To further evaluate the nutritional quality of the microbial lipids, AI, TI, and HH were analyzed. These indices complement compositional parameters by estimating the potential cardiovascular effects of the lipid fraction [58]. Lower AI and TI values are generally associated with higher proportions of unsaturated fatty acids and lower contents of hypercholesterolemic saturated fatty acids such as C12:0, C14:0, and C16:0 [59]. In the present study, AI and TI values ranged from 0.23 to 0.74 and 0.32 to 1.12, respectively. The lowest AI and TI values were generally observed at 15 °C with high C/N ratios, indicating a reduced atherogenic and thrombogenic potential of the produced lipids. This trend was supported by the HH ratio, which ranged from 1.33 to 4.78 and increased with the C/N ratio, particularly at 15 °C, reaching its highest values at C/N 12–14. Since higher HH values reflect a greater proportion of hypocholesterolemic relative to hypercholesterolemic fatty acids, these results suggest that increasing the C/N ratio improved the nutritional quality of the lipid fraction. Overall, the combined behavior of AI, TI, and HH highlights the importance of fatty acid composition in determining the nutritional value of microbial lipids, where higher proportions of MUFA and PUFA relative to SFA are generally considered more favorable [52,58,59].

When all nutritional indices were considered together, the cultivation condition of 15 °C with a C/N ratio of 12 provided the most balanced lipid profile. This treatment combined the highest DU, high DI, the highest PUFA/SFA ratio, low AI and TI values, a high HH ratio, and moderate ω-6/ω-3 and LA/ALA ratios, indicating a favorable balance between lipid unsaturation and nutritional quality. In contrast, although cultivation at 25 °C and C/N 14 resulted in the highest SI and HH values together with a high MUFA/SFA ratio, its lower DU, lower PUFA/SFA ratio, and higher ω-6/ω-3 and LA/ALA ratios suggest a less balanced nutritional profile. Therefore, the optimal cultivation condition depends on the intended application. While cultivation at 15 °C with a C/N ratio of 12 favored the production of a nutritionally balanced lipid, cultivation at 25 °C with a C/N ratio of 14 was more suitable for obtaining a MUFA-enriched oil with a higher oiliness index. These results demonstrate that cultivation conditions can be used to tailor the fatty acid composition of microbial lipids according to specific nutritional or technological objectives.

This ability to modulate lipid composition has important biotechnological implications and can be interpreted within the framework of strategic lipid engineering. Structured lipids represent a technological approach aimed at designing “tailor-made” fats by modifying either the fatty acid composition or their positional distribution on the glycerol backbone [60,61]. This strategy seeks to generate lipids with specific metabolic, cardiovascular, and nutritional functions. Although the present study did not evaluate the positional distribution of fatty acids, the results demonstrate that cultivation conditions can effectively tailor the fatty acid profile and overall nutritional quality of microbial lipids. In this context, *R. mucilaginosa* 6S represents not only an alternative source of microbial oil but also a promising biotechnological platform for producing lipids with targeted compositional and functional properties.

In addition to nutritional quality, oxidative stability should also be considered when developing functional microbial lipids. Although increasing the degree of unsaturation enhances the nutritional value of lipids, it may also increase their susceptibility to oxidative deterioration. In vegetable oils, natural antioxidants such as tocopherols play an important role in preserving oil quality during processing and storage [62,63,64]. In this regard, species of the genus *Rhodotorula* are particularly interesting because they naturally synthesize carotenoids, pigments with antioxidant properties that may help reduce lipid peroxidation and improve oxidative stability. Therefore, future studies should evaluate the oxidative stability of *R. mucilaginosa* 6S lipids and determine the contribution of its endogenous carotenoids, as well as the potential of antioxidant supplementation to further preserve its functional quality [14,65].

## 5. Conclusions

Molasses proved to be a suitable and sustainable substrate for the cultivation of *Rhodotorula mucilaginosa* 6S, supporting biomass and lipid production under low C/N ratio conditions. Biomass formation was mainly influenced by the C/N ratio, whereas temperature strongly modulated lipid accumulation, fatty acid composition, and the nutritional quality of the microbial lipids. Although cultivation at 25 °C improved lipid yield and productivity, 15 °C favored polyunsaturated fatty acid production and better growth kinetics. The nutritional indices confirmed that lipid quality was affected by both temperature and C/N ratio. Overall, 15 °C promoted a more balanced fatty acid profile, with higher MUFA/SFA and PUFA/SFA ratios, and more moderate omega-6/omega-3 and LA/ALA ratios. In contrast, 25 °C mainly favored MUFA accumulation, but with a lower PUFA contribution and less balanced essential fatty acid ratios. Health-related indices, including AI, TI, and HH, also indicated favorable lipid quality, particularly under higher C/N ratios. Considering lipid production, fatty acid profile, and nutritional indices together, cultivation at 15 °C and a C/N ratio of 12 was identified as the most suitable condition for producing microbial lipids with improved nutritional properties. This condition combined high unsaturation, low atherogenic and thrombogenic potential, a favorable HH ratio, high desaturation activity, and the best PUFA/SFA balance. Taken together, these results provide a first approximation to understanding how C/N ratio and temperature modulate the conversion of molasses into biomass and the quality of the resulting microbial lipids. Further optimization, including the evaluation of higher C/N ratios and cultivation strategies such as fed-batch operation, will be necessary to enhance lipid accumulation and assess the feasibility of microbial oil production at an industrial scale. These findings support the potential of *R. mucilaginosa* 6S for the sustainable bioconversion of molasses into microbial lipids enriched in nutritionally relevant unsaturated fatty acids.

## Figures and Tables

**Table 1:** Elemental analysis of the components of the culture medium.

Components of the Culture Medium	Concentrations (g/L)	% C	% N
Molasses	10.00	36.740 ± 1.881	1.735 ± 0.097
	20.00	36.740 ± 0.030	1.736 ± 0.018
	30.00	36.741 ± 0.013	1.735 ± 0.008
	50.00	36.740 ± 0.008	1.734 ± 0.019
	70.00	36.742 ± 0.054	1.735 ± 0.063
	100.00	36.153 ± 0.127	1.69 ± 0.123
Ammonium sulfate	3.75	0.000 ± 0.000	21.18 ± 0.005
Yeast extract	2.00	40.000 ± 0.090	11.000 ± 0.008
Malt extract	1.00	54.000 ± 0.021	5.000 + 0.019

Values are represented with standard deviation (SD).

**Table 2:** Biomass, total lipids, lipids (%), volumetric productivity, biomass/substrate yield (Y_x/s_), lipids/substrate yield (Y_p/s_), values of the experimental design.

Temperature (°C)	C/N Ratio	Final Biomass (g/L)	Final Lipids (g/L)	Lipids (%)	Productivity (mg/Lh)	Y _x/s_	Y _p/s_
25 °C	4	2.738 ± 0.081 ^b^	0.371 ± 0.025 ^a^	13.575 ± 1.353 ^a^	5.155 ± 0.361 ^bc^	0.486 ± 0.027 ^a^	0.067 ± 0.007 ^a^
	6	3.858 ± 0.029 ^a^	0.469 ± 0.066 ^a^	12.171 ± 1.602 ^ab^	6.525 ± 0.507 ^a^	0.486 ± 0.004 ^a^	0.061 ± 0.004 ^a^
	8	3.903 ± 0.022 ^a^	0.436 ± 0.055 ^a^	11.112 ± 1.352 ^bc^	6.026 ± 0.767 ^a^	0.520 ± 0.003 ^a^	0.066 ± 0.009 ^a^
	10	3.792 ± 0.113 ^a^	0.461 ± 0.016 ^a^	12.175 ± 0.055 ^ab^	6.413 ± 0.220 ^a^	0.193 ± 0.007 ^c^	0.024 ± 0.000 ^c^
	12	3.742 ± 0.114 ^a^	0.416 ± 0.055 ^a^	11.098 ± 1.170 ^bc^	5.777 ± 0.783 ^ab^	0.138 ± 0.002 ^d^	0.015 ± 0.001 ^d^
	14	3.023 ± 0.516 ^b^	0.416 ± 0.077 ^a^	13.727 ± 0.236 ^a^	5.671 ± 0.106 ^ab^	0.090 ± 0.018 ^e^	0.013 ± 0.003 ^d^
15 °C	4	3.321 ± 0.071 ^b^	0.325 ± 0.002 ^a^	9.805 ± 0.179 ^cd^	4.407 ± 0.361 ^c^	0.576 ± 0.017 ^a^	0.053 ± 0.008 ^b^
	6	3.933 ± 0.146 ^a^	0.353 ± 0.055 ^a^	8.947 ± 1.120 ^d^	4.899 ± 0.793 ^bc^	0.503 ± 0.017 ^a^	0.045 ± 0.007 ^b^
	8	3.761 ± 0.273 ^a^	0.335 ± 0.045 ^a^	8.877 ± 0.539 ^d^	4.675 ± 0.650 ^bc^	0.326 ± 0.025 ^b^	0.030 ± 0.001 ^c^
	10	3.823 ± 0.015 ^a^	0.300 ± 0.004 ^a^	7.877 ± 0.085 ^e^	4.183 ± 0.062 ^c^	0.199 ± 0.003 ^c^	0.015 ± 0.002 ^d^
	12	3.766 ± 0.095 ^a^	0.328 ± 0.008 ^a^	8.724 ± 0.018 ^d^	4.563 ± 0.105 ^bc^	0.146 ± 0.009 ^d^	0.012 ± 0.001 ^d^
	14	3.740 ± 0.002 ^a^	0.331 ± 0.013 ^a^	8.856 ± 0.343 ^d^	4.600 ± 0.176 ^bc^	0.120 ± 0.000 ^d^	0.009 ± 0.001 ^d^

Mean values and their respective standard deviations are presented. (^a^, ^b^, ^c^, ^d^, ^e^) indicate significant differences between the variables through a Tukey test (*p* < 0.05): same letter, no significant difference, *p* < 0.05; different letters, statistical difference.

**Table 3:** Other quality indices of lipids produced by *Rhodotorula mucilaginosa* 6S at 15 °C and 25 °C.

T	C/N	DU	AI	TI	HH	DI	SI
15 °C	C/N 4	91.25 ± 1.00 ^cd^	0.46 ± 0.03 ^b^	0.55 ± 0.04 ^b^	2.41 ± 0.20 ^d^	0.80 ± 0.00 ^c^	1.41 ± 0.11 ^fg^
	C/N 6	94.61 ± 10.32 ^bcd^	0.43 ± 0.06 ^bc^	0.50 ± 0.02 ^b^	2.78 ± 0.42 ^cd^	0.85 ± 0.01 ^b^	1.79 ± 0.48 ^efg^
	C/N 8	97.82 ± 1.67 ^abcd^	0.42 ± 0.03 ^bcd^	0.46 ± 0.03 ^bcd^	2.61 ± 0.17 ^d^	0.83 ± 0.00 ^bc^	1.42 ± 0.05 ^fg^
	C/N 10	107.36 ± 0.59 ^abc^	0.33 ± 0.00 ^cdef^	0.37 ± 0.03 ^cd^	3.54 ± 0.06 ^bc^	0.93 ± 0.03 ^a^	2.12 ± 0.03 ^ef^
	C/N 12	111.84 ± 6.96 ^a^	0.29 ± 0.03 ^ef^	0.32 ± 0.03 ^d^	3.96 ± 0.34 ^ab^	0.95 ± 0.01 ^a^	2.34 ± 0.18 ^de^
	C/N 14	109.72 ± 2.04 ^ab^	0.28 ± 0.02 ^ef^	0.35 ± 0.02 ^d^	4.00 ± 0.25 ^ab^	0.93 ± 0.00 ^a^	2.41 ± 0.21 ^de^
25 °C	C/N 4	93.20 ± 0.16 ^cd^	0.34 ± 0.02 ^cde^	0.52 ± 0.02 ^b^	3.69 ± 0.25 ^bc^	0.94 ± 0.00 ^a^	3.09 ± 0.17 ^bcd^
	C/N 6	101.72 ± 3.24 ^abcd^	0.32 ± 0.01 ^def^	0.37 ± 0.01 ^cd^	3.92 ± 0.19 ^ab^	0.93 ± 0.00 ^a^	2.93 ± 0.11 ^cd^
	C/N 8	91.01 ± 4.75 ^d^	0.31 ± 0.01 ^ef^	0.49 ± 0.04 ^bc^	3.83 ± 0.12 ^b^	0.96 ± 0.01 ^a^	3.41 ± 0.06 ^bc^
	C/N 10	69.84 ± 0.87 ^e^	0.74 ± 0.04 ^a^	1.12 ± 0.06 ^a^	1.33 ± 0.10 ^e^	0.93 ± 0.01 ^a^	1.20 ± 0.10 ^g^
	C/N 12	86.85 ± 2.08 ^d^	0.27 ± 0.01 ^ef^	0.52 ± 0.03 ^b^	4.13 ± 0.30 ^ab^	0.94 ± 0.00 ^a^	3.86 ± 0.30 ^ab^
	C/N 14	86.98 ± 1.33 ^d^	0.23 ± 0.00 ^f^	0.46 ± 0.01 ^bcd^	4.78 ± 0.09 ^a^	0.94 ± 0.00 ^a^	4.60 ± 0.10 ^a^

DU (Degree of Unsaturation), AI (Atherogenic Index), TI (Thrombogenic Index), HH (Hypocholesterolemic/Hypercholesterolemic Ratio), DI (Desaturation Index), SI (Spreadability Index). Mean values and their respective standard deviations are presented. (^a^, ^b^, ^c^, ^d^, ^e^, ^f^, ^g^) indicate significant differences between the variables through a Tukey test (*p* < 0.05): same letter, no significant difference, *p* < 0.05; different letters, statistical difference.

## Data Availability

The original contributions presented in this study are included in the article/Supplementary Material. Further inquiries can be directed to the corresponding author.

## Supplementary Materials

The following supporting information can be downloaded at: https://www.mdpi.com/article/10.3390/biom16091294/s1, Table S1: Experimental design matrix with standard order; Table S2: Lipid profile of *R. mucilaginosa* at a temperature of 15 °C; Table S3: Lipid profile of *R. mucilaginosa* at a temperature of 25 °C; Table S4: Specific growth rate (µ) under different experimental conditions; Table S5: Determination of total sugars (initial and final) by HPLC.

## References

[1] Valenzuela A., Nieto S. (2003). Ácidos grasos omega-6 y omega-3 en la nutrición perinatal: Su importancia en el desarrollo del sistema nervioso y visual. Rev. Chil. Pediatr..

[2] Marileo L., Acuña J., Rilling J., Díaz P., Langellotti A.L., Russo G.L., Barra P.J., Dantagnan P., Viscardi S. (2023). Protist–Lactic Acid Bacteria Co-Culture as a Strategy to Bioaccumulate Polyunsaturated Fatty Acids in the Protist Aurantiochytrium sp. T66. Mar. Drugs.

[3] Hallahan B., Garland M.R. (2005). Essential fatty acids and mental health. Br. J. Psychiatry.

[4] Minihane A.M., Lovegrove J.A. (2006). 5—Health benefits of polyunsaturated fatty acids (PUFAs). Improving the Fat Content of Foods.

[5] Vásquez-Sandoval C., Navarrete J., Herrera-Herrera P., Dantagnan P., Diaz-Navarrete P., Arancibia-Avila P., Oviedo C. (2023). Screening and Identification of Coastal Chilean *Thraustochytrids* for Arachidonic Acid Production: Biotechnological Potential of *Ulkenia visurgensis* Lng2 Strain. Microorganisms.

[6] Díaz-Navarrete P., Marileo L., Madrid H., Belezaca C., Dantagnan P. (2023). Lipid Production from Native Oleaginous Yeasts Isolated from Southern Chilean Soil Cultivated in Industrial Vinasse Residues. Microorganisms.

[7] Markuš T., Soldat M., Magdevska V., Horvat J., Kavšček M., Kosec G., Petrovič U. (2025). Engineering central metabolism in *Yarrowia lipolytica* increases lipid accumulation. Biochem. Eng. J..

[8] Lopes da Silva T., Fontes A., Reis A., Siva C., Gírio F. (2023). Oleaginous Yeast Biorefinery: Feedstocks, Processes, Techniques, Bioproducts. Fermentation.

[9] Zhang T.T., Wu A.H., Aslam M., Song J.Z., Chi Z.M., Liu G.L. (2024). Developing *Rhodotorula* as microbial cell factories for the production of lipids and carotenoids. Green Carbon.

[10] Díaz-Navarrete P., Marileo L., Madrid H., Mardones W., Correa Galeote D., Parra N., Dehnhardt-Amengual S., Dantagnan P. (2026). Native oleaginous yeasts *Rhodotorula mucilaginosa* and *Solicoccozyma gelidoterrea*: A sustainable biotechnological alternative for lipid production with potential application in diets for farmed fish. Front. Fungal Biol..

[11] Deeba F., Kumar K.K., Wani S.A., Singh A.K., Sharma J., Gaur N.A. (2022). Enhanced biodiesel and β-carotene production in *Rhodotorula pacifica* INDKK using sugarcane bagasse and molasses by an integrated biorefinery framework. Bioresour. Technol..

[12] Harder M., Delabio A., Cazassa S., Remedio R., Pires J., Monteiro T., Arthur V., Mendez-Vilas A. (2013). Lipid production by *Yarrowia lipolytica* for biofuels. Materials and Processes for Energy: Communicating Current Research and Technological Developments.

[13] Li Z., Li C., Cheng P., Yu G. (2022). *Rhodotorula mucilaginosa*—Alternative sources of natural carotenoids, lipids, and enzymes for industrial use. Heliyon.

[14] Diaz-Navarrete P., Sáez A., Marileo L., Alors D., Correa D., Dantagnan P. (2024). Enhancing Selenium Accumulation in *Rhodotorula mucilaginosa* Strain 6S Using a Proteomic Approach for Aquafeed Development. Biomolecules.

[15] Maza D.D., Viñarta S.C., Su Y., Guillamón J.M., Aybar M.J. (2020). Growth and lipid production of *Rhodotorula glutinis* R4, in comparison to other oleaginous yeasts. J. Biotechnol..

[16] Patel A., Pruthi V., Pruthi P.A. (2017). Synchronized nutrient stress conditions trigger the diversion of CDP-DG pathway of phospholipids synthesis towards de novo TAG synthesis in oleaginous yeast escalating biodiesel production. Energy.

[17] Villegas-Méndez M.Á., Montañez J., Contreras-Esquivel J.C., Salmerón I., Koutinas A.A., Morales-Oyervides L. (2023). Scale- up and fed-batch cultivation strategy for the enhanced co-production of microbial lipids and carotenoids using renewable waste feedstock. J. Environ. Manag..

[18] Brenna J.T., Diau G.Y. (2007). The influence of dietary docosahexaenoic acid and arachidonic acid on central nervous system polyunsaturated fatty acid composition. Prostaglandins Leukot. Essent. Fat. Acids.

[19] De Castro A.T., de Souza A.C., Silva C.F., Martinez S.J., Schwan R.F., Dias D.R. (2025). Biotechnological valorization of yeast strains for lipids and carotenoids production from renewable resources. Biocatal. Agric. Biotechnol..

[20] Lakshmidevi R., Ramakrishnan B., Ratha S.K., Bhaskar S., Chinnasamy S. (2021). Valorisation of molasses by oleaginous yeasts for single cell oil (SCO) and carotenoids production. Environ. Technol. Innov..

[21] Ulbricht T.L.V., Southgate D.A.T. (1991). Coronary heart disease: Seven dietary factors. Lancet.

[22] Bansal N., Dasgupta D., Hazra S., Bhaskar T., Ray A., Ghosh D. (2020). Effect of utilization of crude glycerol as substrate on fatty acid composition of an oleaginous yeast *Rhodotorula mucilaginosa* IIPL32: Assessment of nutritional indices. Bioresour. Technol..

[23] Chen J., Liu H. (2020). Nutritional indices for assessing fatty acids: A mini-review. Int. J. Mol. Sci..

[24] Nure J.F., Shibeshi N.T., Asfaw S.L., Audenaert W., Van Hulle S.W. (2017). COD and colour removal from molasses spent wash using activated carbon produced from bagasse fly ash of Matahara sugar factory, Oromiya region, Ethiopia. Water SA.

[25] Griffiths M.J., Harrison S.T. (2009). Lipid productivity as a key characteristic for choosing algal species for biodiesel production. J. Appl. Phycol..

[26] Sluiter A., Hames B., Ruiz R., Scarlata C., Sluiter J., Templeton D. (2006). Determination of sugars, byproducts, and degradation products in liquid fraction process samples. Gold. Natl. Renew. Energy Lab..

[27] Jose J.C., Soares B.C., de Jesus Costa T., Brexo R.P., Thomazini M., Tosi M.M., Favaro- Trindade C.S. (2025). Biosorption in brewer’s spent yeast followed by freeze-drying: A promising strategy to protect vitamin C. LWT.

[28] Dien B.S., Slininger P.J., Kurtzman C.P., Moser B.R., O’Bryan P.J. (2016). Identification of superior lipid producing *Lipomyces* and *Myxozyma* yeasts. AIMS Environ. Sci..

[29] Leyton A., Shene C., Chisti Y., Asenjo J.A. (2022). Production of carotenoids and phospholipids by *Thraustochytrium* sp. in batch and repeated-batch culture. Mar. Drugs.

[30] Morrison W.R., Smith L.M. (1964). Preparation of fatty acid methyl esters and dimethylacetals from lipids with boron fluoride–methanol. J. Lipid Res..

[31] Amza R.L., Kahar P., Juanssilfero A.B., Miyamoto N., Otsuka H., Kihira C., Ogino C., Kondo A. (2019). High cell density cultivation of *Lipomyces starkeyi* for achieving highly efficient lipid production from sugar under low C/N ratio. Biochem. Eng. J..

[32] Danesi E.D.G., Miguel Â.S.M., de Oliveira Rangel-Yagui C., De Carvalho J.C.M., Pessoa A. (2006). Effect of carbon: Nitrogen ratio (C: N) and substrate source on glucose-6-phosphate dehydrogenase (G6PDH) production by recombinant *Saccharomyces cerevisiae*. J. Food Eng..

[33] Reyna-Martínez R., Gomez-Flores R., López-Chuken U.J., González-González R., Fernández-Delgadillo S., Balderas-Rentería I. (2015). Lipid production by pure and mixed cultures production by pure and mixed cultures of *Chlorella pyrenoidosa* and *Rhodotorula mucilaginosa* isolated in Nuevo Leon, Mexico. Appl. Biochem. Biotechnol..

[34] Rosas-Paz M., Zamora-Bello A., Torres-Ramírez N., Villarreal-Huerta D., Romero-Aguilar L., Pardo J.P. (2024). Nitrogen limitation-induced adaptive response and lipogenesis in the Antarctic yeast *Rhodotorula mucilaginosa* M94C9. Front. Microbiol..

[35] Prabhu A.A., Gadela R., Bharali B., Deshavath N.N., Dasu V.V. (2019). Development of high biomass and lipid yielding medium for newly isolated *Rhodotorula mucilaginosa*. Fuel.

[36] Costa W.A.D., Padilha C.E.D.A., Oliveira Júnior S.D.D., Silva F.L.H.D., Silva J., Ancântara M.A., Ferrari M., Santos E.S.D. (2020). Oil-lipids, carotenoids and fatty acids simultaneous production by *Rhodotorula mucilaginosa* CCT3892 using sugarcane molasses as carbon source. Braz. J. Food Technol..

[37] Egli T., Quayle J.R. (1986). Influence of the carbon: Nitrogen ratio of the growth medium on the cellular composition and the ability of the methylotrophic yeast *Hansenula polymorpha* to utilize mixed carbon sources. Microbiology.

[38] Adel A., El-Baz A., Shetaia Y., Sorour N.M. (2021). Biosynthesis of polyunsaturated fatty acids by two newly cold-adapted Egyptian marine yeast. 3 Biotech.

[39] Amaretti A., Raimondi S., Sala M., Roncaglia L., De Lucia M., Leonardi A., Rossi M. (2010). Single cell oils of the cold-adapted oleaginous yeast *Rhodotorula glacialis* DBVPG 4785. Microb. Cell Factories.

[40] Abaza A.A., Shetaia Y.M., Sorour N.M., El-Sayed A.S., El-Baz A.F. (2024). Enhancing the biosynthesis of polyunsaturated fatty acids by *Rhodotorula mucilaginosa* and *Lodderomyces elongisporus*. Bioresour. Bioprocess..

[41] Di Menna M.E. (1966). Yeasts in Antarctic soils. Antonie Leeuwenhoek.

[42] Hu H., Yan F., Wilson C., Shen Q., Zheng X. (2015). The ability of a cold-adapted *Rhodotorula mucilaginosa* strain from Tibet to control blue mold in pear fruit. Antonie Leeuwenhoek.

[43] Parmar T.S., Ahirwar R., Sahay S. (2023). Isolation, purification and characterization of carboxymethyl cellulase (CMCase) from psychrotolerant yeast *Rhodotorula mucilaginosa* BPT1. Mater. Today Proc..

[44] Jin M., Slininger P.J., Dien B.S., Waghmode S., Moser B.R., Orjuela A., da Costa Sousa L., Balan V. (2015). Microbial lipid-based lignocellulosic biorefinery: Feasibility and challenges. Trends Biotechnol..

[45] Liu Z.J., Liu L.P., Wen P., Li N., Zong M.H., Wu H. (2015). Effects of acetic acid and pH on the growth and lipid accumulation of the oleaginous yeast *Trichosporon fermentans*. BioResources.

[46] Probst K.V., Vadlani P.V. (2017). Single cell oil production by *Lipomyces starkeyi*: Biphasic fed- batch fermentation strategy providing glucose for growth and xylose for oil production. Biochem. Eng. J..

[47] Sriwongchai S., Prasongsuk S., Leerat N., Kitleartpornpairoat R. (2018). Possibility of soil mangrove-derived oleaginous yeast *Rhodotorula mucilaginosa* on nutritionally optimized medium for lipid production for alternative biodiesel feedstock. Burapha Sci. J..

[48] Vieira J.P.F., Ienczak J.L., Costa P.S., Rossell C.E.V., Franco T.T., Pradella J.G.C. (2016). Single cell oil production integrated to a sugarcane-mill: Conceptual design, process specifications and economic analysis using molasses as raw material. Ind. Crops Prod..

[49] Abeln F., Chuck C.J. (2020). The role of temperature, pH and nutrition in process development of the unique oleaginous yeast *Metschnikowia pulcherrima*. J. Chem. Technol. Biotechnol..

[50] Redón M., Guillamón J.M., Mas A., Rozès N. (2011). Effect of growth temperature on yeast lipid composition and alcoholic fermentation at low temperature. Eur. Food Res. Technol..

[51] Liszkowska W., Berlowska J. (2021). Yeast fermentation at low temperatures: Adaptation to changing environmental conditions and formation of volatile compounds. Molecules.

[52] Dubois V., Breton S., Linder M., Fanni J., Parmentier M. (2008). Proposition de classement des sources végétales d’acides gras en fonction de leur profil nutritionnel. OCL.

[53] Zhang Z., Liu L., Xie C., Li D., Xu J., Zhang M., Zhang M. (2014). Lipid contents, fatty acid profiles and nutritional quality of nine wild caught freshwater fish species of the Yangtze Basin, China. J. Food Nutr. Res..

[54] Bortoluzzi L., Casal S., Cruz R., Peres A.M., Baptista P., Rodrigues N. (2025). Nutritional and health claims compliance of olive oils produced from centenarian olive trees. Cogent Food Agric..

[55] Visentainer J.V., Santos O.O., Maldaner L., Zappielo C., Neia V., Visentainer L., Pelissari L., Pizzo J., Rydlewski A., Silveira R. (2018). Lipids and fatty acids in human milk: Benefits and analysis. Biochemistry and Health Benefits of Fatty Acids.

[56] Bhattacharya K., Rattan S.I. (2021). Fats and oils for health and longevity. Nutrition, Food and Diet in Ageing and Longevity.

[57] da Rocha K.R., Roesler B.C.S., da Silveira J.M., Burkert C.A.V. (2026). Acid hydrolysis of soybean hulls for lipid production by *Rhodotorula mucilaginosa* CCT 7688. Braz. J. Chem. Eng..

[58] López-Puebla S., Arias-Santé M.F., Romero J., Costa de Camargo A., Rincón-Cervera M.Á. (2025). Analysis of fatty acid profile, α-tocopherol, squalene and cholesterol content in edible parts and by-products of south pacific wild fishes. Mar. Drugs.

[59] Khalili Tilami S., Kouřimská L. (2022). Assessment of the nutritional quality of plant lipids using atherogenicity and thrombogenicity indices. Nutrients.

[60] Osborn H.T., Akoh C.C. (2002). Structured lipids-novel fats with medical, nutraceutical, and food applications. Compr. Rev. Food Sci. Food Saf..

[61] Lopes P.A., Alfaia C.M., Pestana J.M., Prates J.A. (2023). Structured lipids engineering for health: Novel formulations enriched in n-3 long-chain polyunsaturated fatty acids with potential nutritional benefits. Metabolites.

[62] Fernández-Martínez J.M., Pérez-Vich B., Velasco L., Domínguez J. (2007). Breeding for specialty oil types in sunflower. Helia.

[63] Maszewska M., Florowska A., Dłużewska E., Wroniak M., Marciniak-Lukasiak K., Żbikowska A. (2018). Oxidative stability of selected edible oils. Molecules.

[64] Durand E., Laguerre M., Bourlieu-Lacanal C., Lecomte J., Villeneuve P. (2025). Navigating the complexity of lipid oxidation and antioxidation: A review of evaluation methods and emerging approaches. Prog. Lipid Res..

[65] Diaz-Navarrete P., Dantagnan P., Henriquez D., Soto R., Correa-Galeote D., Sáez-Arteaga A. (2024). Selenized non-*Saccharomyces* yeasts and their potential use in fish feed. Fish. Physiol. Biochem..

