# Supplementary material for: Modulation of Polyunsaturated Fatty Acid Production and Nutritional Lipid Quality Indices in Rhodotorula mucilaginosa 6S Using Molasses as a Sustainable Carbon Source
Source: Biomolecules. 2026 Sep 8;16(9):1294. doi: 10.3390/biom16091294 (PMC13604044; doi:10.3390/biom16091294)
Supplement: Supplementary file 1 [file biomolecules-16-01294-s001.zip › biomolecules-4512944-supplementary.pdf]

Supplementary material

Table S1. Experimental design matrix with standard order.

Std	Run	C/N ratio	Temperature (^oC)
1	20	4	15
2	19	4	15
3	11	6	15
4	4	6	15
5	18	8	15
6	2	8	15
7	12	10	15
8	16	10	15
9	7	12	15
10	14	12	15
11	21	14	15
12	6	14	15
13	23	4	25
14	13	4	25
15	22	6	25
16	24	6	25
17	15	8	25
18	8	8	25
19	9	10	25
20	17	10	25
21	10	12	25
22	3	12	25
23	5	14	25
24	1	14	25

Table S2. Lipid profile of *R. mucilaginosa* at a temperature of 15°C

	**Temperature: 15 °C**					
	**C/N 4**	**C/N 6**	**C/N 8**	**C/N 10**	**C/N 12**	**C/N 14**
Saturated Fatty Acids						
C12:0 Lauric Acid	0.00±0.00	0.00±0.00	0.00±0.00	0.55±0.05	0.25±0.08	0.00±0.00
C14:0 Myristic Acid	1.48±0.04	1.97±0.21	1.18±0.25	1.18±0.06	1.12±0.00	0.96±0.08
C15:0 Pentadecanoic acid	0.22±0.02	0.29±0.08	0.28±0.08	0.09±0.04	0.00±0.00	0.08±0.03
C16:0 Palmitic Acid	23.59±2.39	21.08±0.20	23.37±1.99	19.33±0.47	17.88±0.65	17.65±1.25
C17:0 Heptadecanoic acid	0.17±0.03	0.21±0.03	0.00±0.00	0.00±0.00	0.00±0.00	0.00±0.00
C18:0 Stearic Acid	8.16±0.06	6.91±1.34	6.93±0.23	2.99±1.41	2.10±0.33	3.30±0.01
C22:0 Behenic Acid	0.64±0.13	0.27±0.08	0.34±0.08	0.00±0.00	0.00±0.00	0.00±0.00
C23:0 Tricosanoic Acid	0.41±0.01	0.32±0.02	0.28±0.10	0.00±0.00	0.00±0.00	0.00±0.00
C24:0 Lignoceric Acid	1.38±0.13	0.93±0.02	0.73±0.05	0.26±0.10	0.27±0.08	0.18±0.02
**Total of SAFAs**	**36.06±2.81**	**31.97±1.50**	**33.10±2.79**	**24.40±1.81**	**21.62±0.82**	**22.18±1.31**
Monounsaturated Fatty Acids						
C16:1 Palmitoleic Acid	2.07±0.10	2.64±0.86	2.18±0.22	2.55±0.48	2.85±1.05	3.12±0.17
C17:1 Cis-10-Heptadecenoic Acid	0.14±0.03	0.00±0.00	0.00±0.00	0.00±0.00	0.00±0.00	0.00±0.00
C18:1n9c Oleic Acid	33.38±0.88	37.83±9.70	33.15±1.71	40.92±1.49	41.78±1.73	42.49±0.71
C20:1n9 Cis-11-Eicosenoic Acid	0.41±0.03	0.62±0.03	0.46±0.06	0.37±0.09	0.29±0.02	0.30±0.02
C24:1n9 Nervonic Acid	0.64±0.03	0.36±0.02	0.18±0.11	0.00±0.00	0.00±0.00	0.00±0.00
**Total of MUFAs**	**36.63±1.07**	**41.44±10.60**	**35.97±1.66**	**43.84±2.06**	**44.91±2.76**	**45.91±0.52**
Polyunsaturated Fatty Acids						
C18:2n6c Linoleic Acid	15.61±0.08	15.87±0.04	16.39±0.09	21.51±1.16	23.17±1.69	22.56±1.27
C18:3n3	7.74±0.07	6.55±0.15	7.44±0.17	9.61±0.26	10.29±0.41	9.35±0.01

Linolenic Acid						
C18:3n6 G-linolenic acid	0.19±0.02	0.24±0.05	0.00±0.00	0.00±0.00	0.00±0.00	0.00±0.00
C20:4n6 Arachidonic Acid	0.50±0.02	0.39±0.01	0.78±0.01	0.07±0.01	0.00±0.00	0.00±0.00
C20:5n3 Cis-5,8,11,14,17-Eicosapentaenoic Acid	1.41±0.05	2.03±0.04	1.17±0.04	0.00±0.00	0.00±0.00	0.00±0.00
C22:6n3 Cis-4,7,10,13,16,19-Docosahexaenoic Acid	1.86±0.07	1.51±0.05	5.15±0.04	0.58±0.08	0.00±0.00	0.00±0.00
**Total of PUFAs**	**27.31±0.03**	**26.58±0.14**	**30.92±0.00**	**31.76±1.33**	**33.46±2.10**	**31.91±1.28**
**Total Unsaturated Fatty Acids (UNSFA)**	**63.94±1.03**	**68.03±10.46**	**66.90±1.66**	**75.60±0.74**	**78.38±4.86**	**77.82±0.76**
Omega-9	34.42±0.94	38.81±9.74	33.79±1.88	41.29±1.58	42.06±1.71	42.79±0.69
Omega-6	16.30±0.08	16.50±0.07	17.16±0.08	21.58±1.15	23.17±1.69	22.56±1.27
Omega-3	11.00±0.05	10.09±0.06	13.76±0.09	10.18±0.18	10.29±0.41	9.35±0.01

Table S3. Lipid profile of *R. mucilaginosa* at a temperature of 25°C

	**Temperature: 25 °C**					
	**C/N 4**	**C/N 6**	**C/N 8**	**C/N 10**	**C/N 12**	**C/N 14**
Saturated Fatty Acids						
C12:0 Lauric Acid	0.71±0.08	0.21±0.13	0.16±0.01	0.49±0.05	0.00±0.00	0.00±0.00
C14:0 Myristic Acid	1.85±0.21	2.03±0.05	1.48±0.11	2.98±0.11	0.98±0.02	0.93±0.04
C15:0 Pentadecanoic acid	0.22±0.02	0.22±0.01	0.00±0.00	0.00±0.00	0.13±0.02	0.16±0.01
C16:0 Palmitic Acid	17.69±0.91	16.52±0.25	17.90±0.14	31.94±1.51	16.77±1.58	14.93±0.11
C17:0 Heptadecanoic acid	0.00±0.00	0.16±0.02	0.00±0.00	0.00±0.00	0.00±0.00	0.00±0.00
C18:0 Stearic Acid	3.60±0.15	3.85±0.21	2.40±0.67	2.89±0.34	3.91±0.01	4.00±0.06
C22:0 Behenic Acid	0.00±0.00	0.22±0.00	0.00±0.00	1.08±0.12	0.00±0.00	0.00±0.00
C23:0 Tricosanoic Acid	0.22±0.01	0.00±0.00	0.00±0.00	0.00±0.00	0.00±0.00	0.00±0.00
C24:0 Lignoceric Acid	0.00±0.00	0.00±0.00	0.16±0.03	0.48±0.06	0.12±0.04	0.13±0.01
**Total of SAFAs**	**24.31±1.04**	**23.20±0.06**	**22.09±0.72**	**39.86±2.20**	**21.91±1.65**	**20.15±0.01**
Monounsaturated Fatty Acids						
C16:1 Palmitoleic Acid	3.25±1.36	2.93±0.05	3.44±0.70	3.15±0.07	4.44±0.60	3.82±0.13
C18:1n9t Elaidic Acid	0.00±0.00	0.00±0.00	0.00±0.00	9.09±0.27	0.00±0.00	0.00±0.00
C18:1n9c Oleic Acid	54.63±0.11	48.36±1.06	61.14±0.52	38.19±1.28	64.70±1.11	68.69±1.01
C20:1n9 Cis-11-Eicosenoic Acid	0.30±0.01	0.59±0.01	0.22±0.02	0.00±0.00	0.20±0.02	0.22±0.02
**Total of MUFAs**	**58.17±1.24**	**51.88±1.03**	**64.80±1.24**	**50.43±0.94**	**69.33±1.69**	**72.73±1.12**
Polyunsaturated Fatty Acids						
C18:2n6t Linolealidic Acid	0.00±0.00	0.00±0.00	0.00±0.00	0.63±0.00	0.00±0.00	0.00±0.00
C18:2n6c Linoleic Acid	14.95±0.39	15.52±1.00	10.97±1.37	6.15±0.15	7.78±0.16	6.01±0.11
C18:3n3 Linolenic Acid	2.10±0.14	3.94±0.08	1.53±0.27	0.85±0.11	0.80±0.01	0.80±0.06
C18:3n6 G-linolenic acid	0.00±0.00	0.25±0.01	0.00±0.00	0.00±0.00	0.09±0.01	0.00±0.00

C20:2n6 Cis-11,14-Eicosadienoic Acid	0.00±0.00	0.03±0.00	0.00±0.00	0.00±0.00	0.00±0.00	0.00±0.00
C20:3n3 Cis-11,14,17-Eicosatrienoic Acid	0.00±0.00	0.00±0.00	0.00±0.00	0.72±0.03	0.00±0.00	0.00±0.00
C20:3n6 Cis-8,11,14-Eicosatrienoic Acid	0.00±0.00	0.19±0.12	0.00±0.00	0.00±0.00	0.00±0.00	0.00±0.00
C20:4n6 Arachidonic Acid	0.00±0.00	0.34±0.01	0.00±0.00	1.36±0.04	0.00±0.00	0.00±0.00
C20:5n3 Cis-5,8,11,14,17-Eicosapentaenoic Acid	0.00±0.00	2.49±0.02	0.29±0.09	0.00±0.00	0.00±0.00	0.14±0.13
C22:6n3 Cis-4,7,10,13,16,19-Docosahexaenoic Acid	0.46±0.01	2.14±0.11	0.31±0.03	0.00±0.00	0.10±0.02	0.17±0.03
**Total of PUFAs**	**17.51±0.54**	**24.92±1.11**	**13.10±1.76**	**9.70±0.03**	**8.76±0.20**	**7.12±0.10**
**Total Unsaturated Fatty Acids (UNSFA)**	**75.69±0.70**	**76.80±2.13**	**77.91±2.99**	**60.14±0.91**	**78.09±1.89**	**79.85±1.22**
Omega-9	54.93±0.12	48.95±1.08	61.36±0.54	47.29±1.01	64.89±1.09	68.91±0.99
Omega-6	14.95±0.39	16.34±0.90	10.97±1.37	7.51±0.11	7.86±0.17	6.01±0.11
Omega-3	2.56±0.15	8.57±0.21	2.14±0.39	1.56±0.13	0.90±0.02	1.11±0.01

Table S4. Specific growth rate (μ) under different experimental conditions.

Temperature (°C)	C/N ratio	μ (h^{-1})
25 °C	4	0.042 ± 0.001^{c}
	6	0.046 ± 0.003^{c}
	8	0.054 ± 0.002^{ab}
	10	0.056 ± 0.003^{ab}
	12	0.056 ± 0.001^{ab}
	14	0.050 ± 0.002^{b}
15 °C	4	0.051 ± 0.001^{b}
	6	0.054 ± 0.003^{ab}
	8	0.059 ± 0.004^{a}
	10	0.059 ± 0.001^{a}
	12	0.059 ± 0.006^{a}
	14	0.058 ± 0.004^{a}

Mean values and their respective standard deviations are presented. (a, b, c) Indicates significant differences between the variables through a Tukey test (p <0.05). Same letter (no significant difference, p <0.05). Different letters (statistical difference).

Table S5. Determination of total sugars (initial and final) by HPLC.

Hours	C/N ratio and temperature	Sucrose (g/L)	Glucose (g/L)	Fructose (g/L)	Arabinose (g/L)	Total Sugars(g/L)
0h	C/N:4; T:15	3.645	0.977	0.069	-	4.691
0h		4.013	0.637	0.042	-	4.692
72h		0.064	0.005	0.000	-	0.069
72h		0.111	0.029	0.001	-	0.141
0h	C/N:6; T:15	5.184	1.850	0.081	-	7.115
0h		5.747	1.372	0.065	-	7.183
72h		0.464	0.109	0.001	-	0.574
72h		0.332	0.261	0.003	-	0.595
0h	C/N:8; T:15	8.935	1.667	0.067	-	10.669
0h		8.501	1.942	0.084	-	10.527
72h		0.690	0.430	0.002	-	1.122
72h		0.598	0.322	0.002	-	0.923
0h	C/N:10; T:15	15.848	2.881	0.095	-	18.824
0h		15.924	2.507	0.086	-	18.517
72h		1.763	0.535	0.002	-	2.300
72h		1.220	0.735	0.000	-	1.956
0h	C/N:12; T:15	23.000	2.482	0.081	-	25.563
0h		22.061	2.851	0.094	-	25.006
72h		2.370	0.690	0.002	-	3.063
72h		2.688	0.821	0.003	-	3.512
0h	C/N:14; T:15	24.414	6.156	0.185	-	30.755
0h		24.141	6.467	0.192	-	30.799
72h		3.036	1.231	0.004	-	4.271
72h		3.141	1.125	0.005	-	4.272
0h	C/N:4; T:25	3.674	0.903	0.061	-	4.638
0h		2.870	1.655	0.117	-	4.642
72h		0.041	0.007	0.000	-	0.048
72h		0.039	0.008	0.000	-	0.047
0h	C/N:6; T:25	5.879	1.235	0.075	-	7.189
0h		5.259	1.668	0.096	-	7.023
72h		0.153	0.036	0.104	-	0.293
72h		0.087	0.066	0.082	-	0.234
0h	C/N:8; T:25	7.512	1.737	0.109	-	9.357
0h		7.566	1.821	0.116	-	9.504
72h		2.555	0.175	0.183	-	2.912
72h		2.521	0.295	0.146	-	2.962
0h	C/N:10; T:25	15.683	2.461	0.131	-	18.275
0h		15.976	2.409	0.111	-	18.496
72h		1.100	0.309	0.027	-	1.436
72h		1.311	0.344	0.002	-	1.657
0h	C/N:12; T:25	22.896	2.641	0.123	-	25.660

0h		23.537	2.245	0.087	-	25.868
72h		1.898	0.398	0.013	-	2.309
72h		2.657	0.461	0.001	-	3.119
0h	C/N:14; T:25	24.690	5.572	0.221	-	30.483
0h		24.467	5.769	0.223	-	30.459
72h		3.202	0.616	0.018	-	3.836
72h		3.019	0.698	0.003	-	3.720
